# Life History of the Arctic Squid *Gonatus fabricii* (Cephalopoda: Oegopsida) Reconstructed by Analysis of Individual Ontogenetic Stable Isotopic Trajectories

**DOI:** 10.3390/ani12243548

**Published:** 2022-12-15

**Authors:** Alexey V. Golikov, Filipe R. Ceia, Hendrik J. T. Hoving, José P. Queirós, Rushan M. Sabirov, Martin E. Blicher, Anna M. Larionova, Wojciech Walkusz, Denis V. Zakharov, José C. Xavier

**Affiliations:** 1 GEOMAR Helmholtz Centre for Ocean Research Kiel, 24105 Kiel, Germany; 2 University of Coimbra, MARE—Marine and Environmental Sciences Centre/ARNET—Aquatic Research Network, Department of Life Sciences, 3000-456 Coimbra, Portugal; 3 British Antarctic Survey, Natural Environment Research Council, Cambridge CB3 0ET, UK; 4 Department of Zoology, Kazan Federal University, 420008 Kazan, Russia; 5 NIRAS A/S, 8000 Aarhus, Denmark; 6 Greenland Climate Research Center, Greenland Institute of Natural Resources, 3900 Nuuk, Greenland; 7 Fisheries and Oceans Canada, Winnipeg, MB R3T 2N6, Canada; 8 Laboratory of Marine Research, Zoological Institute of Russian Academy of Sciences, 199034 Sankt-Petersburg, Russia

**Keywords:** deep-sea, ecology, *δ*^13^C, *δ*^15^N, mixing model, isotopic niche, allometric equation, generalist, specialist, beaks

## Abstract

**Simple Summary:**

*Gonatus fabricii* is the most abundant cephalopod in the Arctic and northern part of the North Atlantic, which are areas of rapid environmental change. It is very important as a predator of many species of fish and invertebrates and as a prey for larger fish, seabirds, and marine mammals. The life cycle of *G. fabricii*, in particular ontogenetic changes in diet and habitat, is little known. Ecological modelling is an important method to forecast ecosystems’ dynamics in relation to a changing climate, and it is crucial to assess their present structure and functioning for a viable forecast. Here, the ontogenetic changes in the diet and habitat of large *G. fabricii* from West Greenland are studied using stable isotope analysis of carbon (*δ*^13^C) and nitrogen (*δ*^15^N) along trajectories in chitin beaks, which function as archival structures. Using this approach, four stages with clearly distinct ecologies were revealed in the species’ life cycle. This novel ecological periodization is a crucial baseline tool in Arctic marine ecosystem studies and cephalopod biology, and should be used in models to correctly reflect the ecological roles of *G. fabricii* in marine ecosystems.

**Abstract:**

Cephalopods are important in Arctic marine ecosystems as predators and prey, but knowledge of their life cycles is poor. Consequently, they are under-represented in the Arctic ecosystems assessment models. One important parameter is the change in ecological role (habitat and diet) associated with individual ontogenies. Here, the life history of *Gonatus fabricii*, the most abundant Arctic cephalopod, is reconstructed by the analysis of individual ontogenetic trajectories of stable isotopes (*δ*^13^C and *δ*^15^N) in archival hard body structures. This approach allows the prediction of the exact mantle length (ML) and mass when the species changes its ecological role. Our results show that the life history of *G. fabricii* is divided into four stages, each having a distinct ecology: (1) epipelagic squid (ML < 20 mm), preying mostly on copepods; (2) epi- and occasionally mesopelagic squid (ML 20–50 mm), preying on larger crustaceans, fish, and cephalopods; (3) meso- and bathypelagic squid (ML > 50 mm), preying mainly on fish and cephalopods; and (4) non-feeding bathypelagic gelatinous females (ML > 200 mm). Existing Arctic ecosystem models do not reflect the different ecological roles of *G. fabricii* correctly, and the novel data provided here are a necessary baseline for Arctic ecosystem modelling and forecasting.

## 1. Introduction

The Arctic Ocean is experiencing the world’s fastest climate change-induced warming [1,2]. This warming is causing changes in different levels of the Arctic marine ecosystems [3,4,5]. Ice retreat as a result of warming allows marine fisheries to move further north to new ice-free Arctic areas [6], which causes anthropogenic disturbance in these vulnerable ecosystems. The need to predict ecosystem changes is reflected in eight (out of 15 total) main principles of the foremost international approach in marine ecosystem-based management [7]. Data on ecosystem structure, biodiversity, species interactions with each other and the environment, and the state of key abiotic parameters from both monitoring and experimental approaches are crucial for ecological forecasting [8]. Ecosystem modelling is a powerful tool for predicting near-future ecosystem change [8]. However, the quality of the models depends on the available input data of the ecosystems’ components. Crucial life history data are lacking for many ecologically important invertebrates in the Arctic Ocean [9], and consequentially, these taxa are scarcely represented in ecosystem models [10,11,12,13,14,15,16,17,18,19,20]. Moreover, invertebrates constitute most of the biodiversity in marine ecosystems, they are a common link between primary producers and secondary consumers, and many invertebrate taxa are sensitive to climate change [9,21,22,23,24].

Cephalopoda (Phylum Mollusca) are an abundant group of marine invertebrates in Arctic marine ecosystems, are known to respond rapidly to environmental change, and have a pivotal role in the food web [23,25,26,27,28,29]. Eleven species of cephalopods complete their life cycle in the Arctic [23,30], and they are ecologically important due to their high abundances and biomasses [29,31,32]. For example, cirrate octopod density can reach up to 94 ind. km^−2^ in the Baffin Bay [29], and squid cumulative biomass is estimated up to 20 million t in the Nordic Seas [31]. They prey on many pelagic and benthic invertebrates and fish and are key prey for top predators such as larger fish, seabirds, and marine mammals [23,26,27,28,29,31,32]. Additionally, Arctic cephalopods occupy various ecological niches and habitats, i.e., pelagic, benthic, nekton, nekto-benthic, and bentho-pelagic [23,25]. Despite their importance, Arctic cephalopods are either missed or poorly incorporated into Arctic biodiversity assessment reports [9,21,22] and Arctic food web models [10,11,12,13,14,15,16,17,18,19,20]. Among the main reasons for this is that many cephalopods are not strictly benthic or pelagic and change habitat during ontogenesis [25,27], and hence have not been included in dedicated Arctic benthos nor nekton reviews [9,22,24]. Furthermore, scarce knowledge of the life history (in particular, their ontogenetic diet and habitat changes, growth patterns, age, and reproduction) of oceanic cephalopods also precludes their higher representation in these models [10,11,12,13,14,15,16,17,18,19,20]. To obtain representative and accurate models, life history parameters need to be obtained for key species. One such key species is *Gonatus fabricii* (Lichtenstein, 1818) [33], the most abundant cephalopod in the Arctic Ocean [31,32,34,35].

*Gonatus fabricii* is the only squid that spends its entire life cycle, including reproduction, in the Arctic [23,25,32,36,37]. It is by far the most abundant cephalopod species in the Arctic Ocean, reaching a cumulative biomass of up to 20 million t in the Nordic Seas, and it is well represented in the deep scattering layer under the ice in the high-latitude Arctic [31,32,34,35]. This species undergoes a continuous ontogenetic descent from epi- to bathypelagic layers, and has diurnal vertical migrations [36,38,39,40,41,42]. There is also a shift in the feeding behavior and diet of *G. fabricii* throughout ontogeny [42,43,44,45,46]. Stomach content analyses of *G. fabricii* show ontogenetic changes in the diet of individuals from the Nordic Seas and adjacent south-west margin of the Barents Sea [43,44,45,46], but not in West Greenland, most likely due to the sampled squid size distribution being biased towards smaller individuals [36]. The food spectrum of *G. fabricii* includes at least 49 taxa (predominantly crustaceans and fish), and *G. fabricii* is preyed upon by 47 taxa (predominantly fish and marine mammals) [27].

Stable isotope signals of *δ*^13^C and *δ*^15^N in hard body structures with continuous growth have been analysed to understand individual variability in feeding over the life history of individual cephalopods e.g., [47,48,49,50,51,52]. Hard body structures of cephalopods can be used to decipher life history and trophic signals of individuals since they are present when the individual hatches and grow for its entire life. Examples of such structures are statoliths, gladii, eye lenses, and beaks [53,54,55]. Upper and lower beaks are chitinous structures in the cephalopod mouth, which might be used for species identification and are well preserved in predators’ stomach contents [56,57,58,59,60]. Moreover, it is possible to estimate individual size, both mantle length (ML) and mass, from beak measurements [56,58].

Beaks are known to grow along their posterior border, where the most recent material is deposited, while the oldest material is deposited in the anterior tip [61,62,63,64,65]. The most anterior part of the beak, the tip of the rostrum, represents the earliest ontogenetic stages, and the most posterior, end of the hood and crest in the upper beak and the end of the hood and crest and wing in the lower beak, represents the latest [47,51,52,66]. The tip of the rostrum is subjected to erosion and new beak material is deposited there throughout the individual’s life [51,62,63]. Thus, values from the earliest ontogenetic stages are influenced by beak layers deposited during the later life periods [51]. Previous studies showed that new beak material is deposited every day in five species of squids and three species of octopods and in different areas of their beaks: see the recent review by Xavier et al. [65]. However, new beak material deposition potentially depends on various factors, especially in deep-sea and cold-water species [65,67,68]. Darkening of the beaks proceeds from the anterior part towards the posterior, as the beaks grow [51,56,58,61]. Tanned parts are very stiff and differ in composition from the semi-liquid transparent posterior-most parts [51,61,69]. This stiffness is achieved by increasing the content of hydrophobic histidine-rich proteins, which causes dehydration and sclerotization of the tissues [70,71]. Thus, the tanned parts of the beaks are metabolically inert [47,51,52,61,65,66,69,70,71]. They are known to lose water, but not any other substances, during their progressive sclerotization [70,71]. Stable isotope signals of *δ*^13^C and *δ*^15^N in the sclerotized tanned parts of the beaks from the experimental study reflected the several months’ past diet of the captive cephalopods, while soft transparent parts of their beaks reflected the recent diet [72]. Thus, the most anterior and posterior parts of the beaks warrant certain methodological considerations (see Materials and Methods) when applying stable isotope analysis (SIA) techniques to different parts of the same beak.

Carbon (*δ*^13^C) and nitrogen (*δ*^15^N) are the most frequently used stable isotopes to investigate trophic relationships in marine food webs [73,74]. Stable isotope analysis has been used on cephalopods to reveal their foraging habitat changes, migrations, and dietary changes throughout their ontogeny e.g., [30,47,50,51,52,75,76]. Specific SIA studies on *G. fabricii* with different ML demonstrated an increase of *δ*^15^N values from small to large individuals, suggesting a change in the food spectra from low to high trophic level (TL) prey as squid grow [27,77]. The combination of ontogenetic descent to deep waters, changes in diet, and an increase in TL suggests that *G. fabricii* occupies several ecological roles during its life-time. However, this hypothesis remains untested. Studies to date have used snapshot data from multiple individuals of different sizes to generalize a pattern over the lifetime of an average individual [27,77]. However, such an approach is incomplete and does not take into account individual variation linked to continuous growth e.g., [50,51,52], and consequently is not really suitable for highlighting potential migrations or clearly defining ontogenetic stages during a lifetime. Thus, the analysis of ontogenetic change in habitat and TL within individual *G. fabricii* is still a drawback in our understanding of the ecology of this key species.

This study tests the following hypotheses: (1) ontogenetic change in habitat use, TL, and diet determined using SIA of multiple individuals of *G. fabricii* is reflected in the stable isotope signal along a trajectory (with continuous growth) in individual beaks; (2) substantial variation exists among *G. fabricii* individuals and between sexes in stable isotope trajectories in beaks; and (3) size-specific changes in habitat, TL, diet, and isotopic niche can be determined via analyses of *δ*^13^C and *δ*^15^N in individual beaks, allowing us to define the species’ ecological roles and their transitions. We strive to provide baseline data on cephalopod biology and in Arctic marine ecosystem studies where *G. fabricii* plays an important role in the structure of the trophic webs and function within the communities. These data can be directly used in ecosystem modelling. Finally, we discuss the use of beaks and other cephalopod hard structures for SIA and provide guidelines and methodological recommendations on how to apply the combination of population and individual SIA to other cephalopods.

## 2. Materials and Methods

### 2.1. Material and Data Used, and Beak Measurements

**Figure 1:**
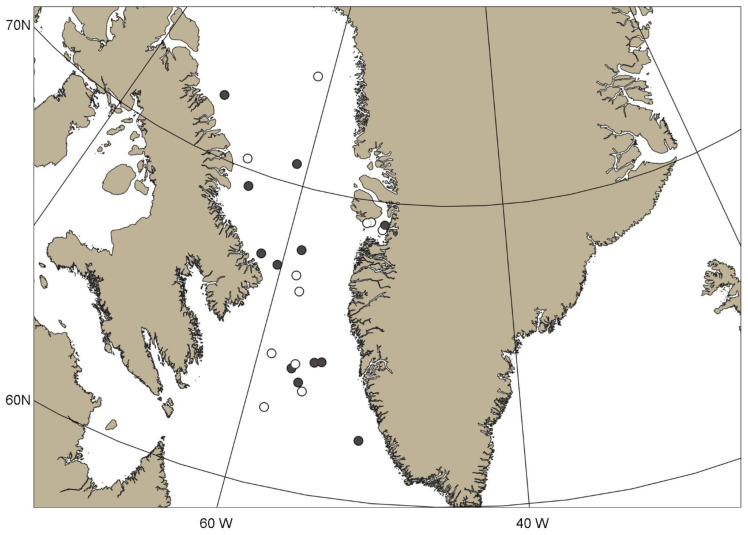
Sampling locations of *Gonatus fabricii* in West Greenland. Filled circles: stations where morphological beak measurements and stable isotope analysis were performed. Empty circles: stations where only morphological beak measurements were performed.

**Figure 2:**
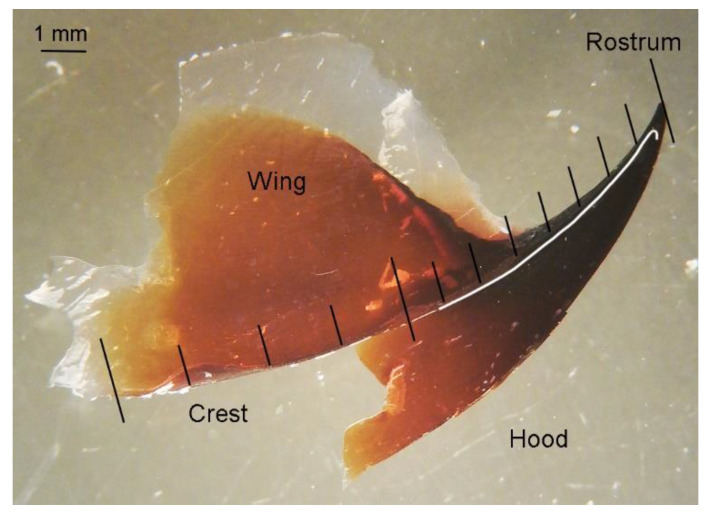
Scheme of the upper beak subsections cut in *Gonatus fabricii* from inside the crest: subsections from the rostrum to the mid-point of the crest = 1 mm, and subsections from the mid-point of the crest to the transparent part of the hood = 2 mm. Upper beak = individual M1 with mantle length 216 mm. The white line shows the crest outline from the inside.

Twenty nine *G. fabricii* individuals with ML > 200 mm were sampled in late June–early November 2016 (R/V ‘Paamiut’, Greenland Institute of Natural Resources, Greenland, and Department of Fisheries and Oceans, Canada), 2017 (R/V ‘Paamiut’, Greenland Institute of Natural Resources) and 2019 (F/V ‘Helga Maria’, Greenland Institute of Natural Resources) in West Greenland (Figure 1, Table S1). The samples were collected as bycatch by bottom trawl ‘Alfredo 3’ at depths of 384.5–1443.0 m (Table S1), and fixed in 4% formalin onboard. The squid’s ML and mass were measured in the laboratory and the sex and maturity stages were identified following Golikov et al. [37]. Afterward, upper beaks were taken and their upper beak rostrum length (URL) and upper beak crest length (UCL) were measured following Clarke [56,58]. A total of fourteen individuals (six females and eight males) were used to obtain ontogenetic stable isotope trajectories. Transparent parts of the upper beaks were cut out as they have significantly different isotopic values from tanned beaks [61,69]. The upper beaks were divided in half along the crest. Sequential equal-sized subsections as close to 1 mm as possible were taken from the inner side of the crest, starting from the rostrum to the mid-point of the crest, and sequential equal-sized subsections as close to 2 mm as possible were taken starting from the midpoint of the crest to the onset of the now-cut transparent part (Figure 2, Table S2). The higher resolution for the earlier half of ontogenesis is explained by the fact that this is where the main changes in isotopic values occurred in other cephalopods [47,51,52]. The tip of the rostrum, which is known to have not only the newest material deposited due to beak growth specifics [51,62,63], represented half of subsection 1 or less with this approach (Figure 2) and it did not bias the outcome (see Results). With 11 to 17 subsections from each upper beak, SIA was performed in 179 samples (Table 1 and Table S2). The upper beak allows a sufficient number of subsections to be analysed in an ontogenetic perspective according to Guerra et al. [47] and Queirós et al. [52] (one study also exists on lower beaks, but only four individuals were analysed [66]). Thus, the upper beak provides a more direct comparison with existing studies where a sufficient number of subsections were analysed. A, lower beaks of *G. fabricii* are much smaller than in other cephalopods, analysed with this approach.

Data from Golikov et al. [27], where 185 beaks of *G. fabricii* were measured and 105 beaks underwent SIA, were used in this study. Beak URL measurements from this study and Golikov et al. [27] were pooled to update the existing equations, allowing us to estimate ML and mass of *G. fabricii* in West Greenland and the Arctic in general: West and East Greenland, and the Barents Sea (Table S3). Beak UCL measurements of individuals from Golikov et al. [27] were not used in the respective study, and they are used here for the first time. The difference between the isotopic composition of upper and lower beaks is significant, but it is small and is within the measurement error of the method [69]. However, this should be kept in mind when the data from this study were united with those from Golikov et al. [27] for SIBER analyses (see below). In Golikov et al. [27], the individuals were grouped as ‘epipelagic’, ‘mesopelagic’, and ‘bathypelagic’ based on ML and maturity stages combined: ML < 60 mm in both sexes, ML 60–130 mm in males and 60–150 mm in females, and ML > 130 mm in males and >150 mm in females. Thus, they are referred to as small, medium, and large here, respectively.

### 2.2. Stable Isotope Analyses

All beak subsections were dried at 60 °C for 24–48 h, weighed (to the closest value to 0.3 mg) with a micro-balance and cracked if needed, and sterile-packed in tin containers. Stable isotopic signatures were determined by a Flash EA 1112 Series elemental analyser (Thermo Scientific Inc., Waltham, MA, USA) coupled online via a Finnigan ConFlo II interface to a Delta vs. mass spectrometer (Thermo Scientific, Bremen, Germany) and expressed as: *δ*^13^C and *δ*^15^N = [(R_sample_/R_standard_) − 1] * 1000, where R = ^13^C/^12^C and ^15^N/^14^N, respectively. The carbon and nitrogen isotope ratios were expressed in delta (*δ*) notation relative to Vienna-PeeDee Belemnite limestone (V-PDB) for *δ*^13^C and atmospheric nitrogen (AIR) for *δ*^15^N. Replicate measurements of internal laboratory standards (acetanilide STD: Thermo Scientific PN 338 36700) in every batch (*n* = 14) indicated a precision <0.2‰ for both *δ*^13^C and *δ*^15^N signatures. The mass C:N ratio for each sample, where it was obtained, ranges from 3.05 to 3.86 (Table S2). The analyses were carried out at the Marine and Environmental Science Centre, University of Coimbra (Coimbra, Portugal).

### 2.3. Data Analyses

The newly obtained equation to estimate ML from UCL was used to find squid ML at given subsections, and 4.2 mm was added to this value (see Beak equations in Results). Squid mass at a given subsection was estimated based on the length-mass equation from Golikov et al. [27].

A regression analysis was used to find equations fitting our data [78]. Correlations between *δ*^13^C and *δ*^15^N values were assessed using a Spearman’s rank correlation [78]. At the population level, *n* = 118 overall and *n* = 57 in West Greenland were used for this analysis. At the individual level, it was possible to assess 7 of 14 individuals, as the rest had missing values for some subsections (Table S2). A Mann–Whitney *U* test was used to compare isotopic signatures of *δ*^13^C and *δ*^15^N and TL between two groups (e.g., sexes), and a Kruskal–Wallis *H* test (with a post-hoc Dunn’s *Z* test) was used to compare among more than two groups (e.g., size groups) [78]. Differences in *δ*^13^C values, *δ*^15^N values, and TL among beak subsections were tested using a Skillings–Mack test proceeded by the Nemenyi post hoc test [79]: only the subsections common to all the beaks, i.e., until the last subsection of the beak with the lowest number of subsections overall, were tested. The packages PMCMR 4.4 [80] and Skillings.Mack 1.10 [81] in R 4.1.3 [82] were used for these analyses. The value of *α* = 0.05 was considered significant in this study.

Stable isotopic values were used to calculate the specialization index (*s*) to estimate the degree of individual specialization [83,84] based on the isotopic variation within and between individuals of a sampled population [85]. The specialization index *s* was defined as the variance of the isotopic signal along the crest of the given individual divided by the total variance among individuals of the sampled population plus the variance of the isotopic signal along the crest of the given individual [85]. Accordingly, *s* values range from 0 to 1, with 1 representing a complete overlap between the individual and the population (= extreme generalist individual) and lower *s* values representing higher degrees of specialization [85]. Individuals occupying < 50% of the total niche of the sampled population (i.e., *s* < 0.5) are considered specialists [85,86]. The specialization index *s* was calculated separately for each individual and for each isotope, *δ*^13^C and *δ*^15^N.

Neither ethanol nor formalin fixation significantly affect *δ*^13^C or *δ*^15^N signatures of cephalopod beaks [87], thus, no corrections were performed due to fixation. Values of *δ*^15^N in cephalopod beaks, in contrast to *δ*^13^C values, are on average 4.8‰ lower than values from muscle tissue [72,75,87,88]. Therefore, when estimating TL, we added 4.8‰ to raw beak *δ*^15^N values as proposed by Cherel et al. [88] and Golikov et al. [27,28,30,76].

Trophic level can be estimated with fixed trophic enrichment factor (TEF), either ‘classical’ *δ*^15^N = 3.4‰ [89] or ‘Arctic’ *δ*^15^N = 3.8‰ [90] and with the standard TL equation [91] or with the scaled TEF equation [92,93] adapted for the Arctic by Linnebjerg et al. [94]. We used the standard TL equation with ‘Arctic’ TEF as the scaled approach was giving unrealistically high results (not included in this paper). We used *Calanus finmarchicus* (Gunnerus, 1770) [95] taken from the nearest station in Hansen et al. [96] or, alternatively, in Pomerleau et al. [97], as a reference value for TL = 2.0 when a closer location was not present in Hansen et al. [96]. Interpretation of TL in the Arctic ecosystems followed the TL interpretations of other stable isotope assessments of the area [27,28,30,90,94,98,99,100,101].

Sequential subsections of the same beak do not comply with the assumption of sample independence, and due to that, nicheROVER 1.1.0 [102] in R 4.1.3 [82] was employed to estimate isotopic niche width for each subsection and overlap among them. These analyses were only applied until subsection 14, as an insufficient number of individuals were large enough to sample subsections 15 (*n* = 6), 16, and 17 (both *n* = 2) (Table 1). It was assumed that the mean isotopic value of all subsections of the given individual corresponded to the isotopic values of the individual obtained by the ‘classical’ approach, i.e., powdering the whole beak and taking a random portion. Thus, the mean isotopic values of beaks from this study (*n* = 14) were pooled with the dataset from Golikov et al. [27], and SIBER 2.1.6 [103] in R 4.1.3 [82] was employed to assess isotopic niche width and overlap between sexes and squid with ML 20–50 mm and ML > 50 mm. Squid with ML < 20 mm (*n* = 4) from West Greenland from Golikov et al. [27] could not be used in this ontogenetic niche comparison due to the low sample number. The standard ellipse area corrected for small sample sizes (SEA_c_) and the Layman metric of convex hull area (TA) were estimated [103,104,105], and the Bayesian approximation of the standard ellipse area (SEA_b_) was employed to compare niche width [103]. The overlap interpretation for both niche-related packages followed Langton [106], where overlap ranging from 0 to 0.29 indicated no overlap, from 0.30 to 0.60 indicated medium overlap, and from 0.61 to 1 indicated large overlap, with only the latter taken as significant [30]. Trophic levels were used instead of *δ*^15^N values (*y* axis) in niche estimations by both niche-related packages. This approach improves the ecological meaning of isotopic data when comparing individuals from different areas and ecosystems as it is a means to counter high *δ*^15^N baseline variation e.g., [107,108]. This approach has been repeatedly applied to cephalopods [28,30,76].

The Bayesian mixing model SIMMR 0.4.5 [109] in R 4.1.3 [82] was used to assess the relative contribution of prey to the diet of *G. fabricii*. Based on an overview of stomach content analyses [27], the prey groups assessed were copepods, euphasiids, shrimps (these three taxa belong to Crustacea), chaetognaths, cephalopods (*G. fabricii*, as it is the only squid species living permanently in the Arctic) and fish. Source isotopic values for the diet model (terminology for the diet model followed its author [109]) were taken from Pomerleau et al. [97,110], Hansen et al. [96], Agersted et al. [111], Linnebjerg et al. [94], Golikov et al. [27], Grigor et al. [101] and this study (i.e., mean values of each studied squid), and are detailed in Table S4. Crustacean and chaetognath source values were similar for all analysed upper beak subsections of *G. fabricii*, and source values of cephalopods and fish were taken according to squid size for ecological perception. For subsections 1–3, cephalopods and fish were combined, as their source values were not significantly different: all source values should be significantly different in at least one of the isotopes (Tables S4 and S5). Values and standard deviations of TEF were taken from the only experimental study showing isotopic values in cephalopods to change following the long-time diet composition [72]: *δ*^13^C = −0.20 ± 0.55‰ and *δ*^15^N = 3.37 ± 0.99‰. The data fitting to selected prey source values and TEFs were checked inside SIMMR 0.4.5 [109] prior to the analysis, and only the fitting values were used in the models (Figure S1), as follows: subsections 1–3, *n* fitting = 23 of 31; subsections 4–5, *n* fitting = 16 of 25; subsections 7–9, *n* fitting = 20 of 36; and subsection 10, *n* fitting = 5 of 10. To compare the results of the models among the subsections, the *χ*^2^ test and Fisher’s exact test were used: although the latter is more adequate for small sample sizes, Fisher’s exact allows the comparison of only two groups [78]. Statistical analyses were performed in R 4.1.3 [82], Statistica 10.0 (Statsoft), and PAST 4.02 [112]. Values are presented as the mean ± SE unless otherwise stated.

## 3. Results

### 3.1. Stable Isotopic Values and Trophic Levels: Ontogenetic Changes

**Figure 3:**
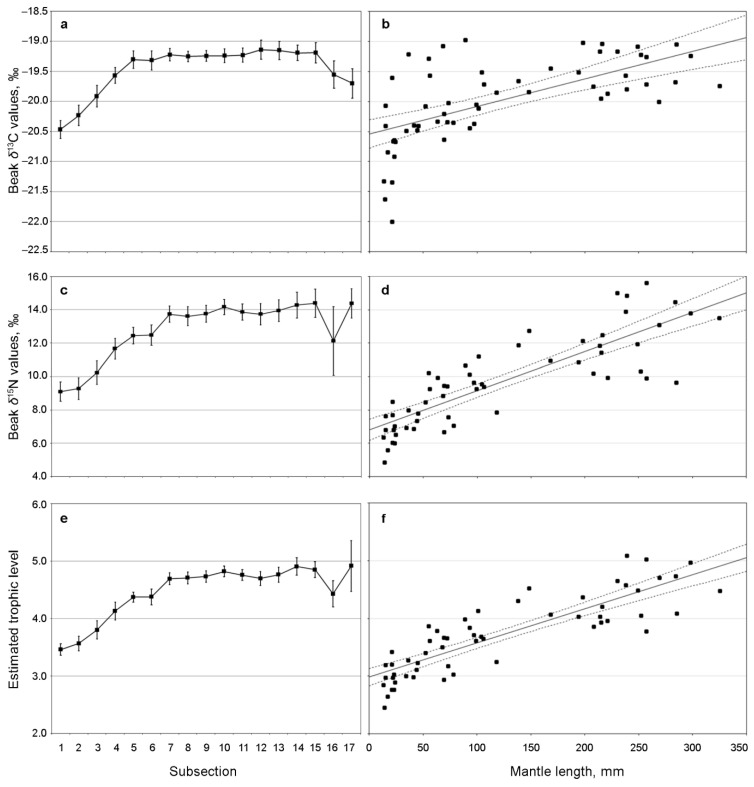
Ontogenetic changes in *δ*^13^C and *δ*^15^N values, and trophic level (TL) in *Gonatus fabricii* from West Greenland at the individual (**a**,**c**,**e**) and population (**b**,**d**,**f**) levels: (**a**) *δ*^13^C, individual level, (**b**) *δ*^13^C, population level, (**c**) *δ*^15^N, individual level, (**d**) *δ*^15^N, population level, (**e**) TL, individual level, and (**f**) TL, population level. Filled squares represent mean value at given subsection (**a**,**c**,**e**) or individual values (**b**,**d**,**f**). Bars (**a**,**c**,**e**) represent SE.

Each of the studied individuals demonstrated an ontogenetic increase of both isotopes (Figure 3, Table 2 and Table 3). Within-individual ontogenetic increase is overall lower than within-population ontogenetic increase: 0.7–2.2‰ (1.4 ± 0.13) vs. 3.0‰ in *δ*^13^C and 2.6–9.1‰ (5.6 ± 0.37) vs. 10.7‰ in *δ*^15^N, respectively. The first subsection (rostrum tip) usually had the lowest value. However, the last subsection (posterior end of the crest), except for the single analysed spent female (F6), never had the highest value. The main increase of *δ*^13^C values was from subsections 1 to 5 with a mean of 0.29‰ per subsection, which decreased to a mean of 0.01‰ per subsection afterward (Figure 3, Table 2 and Table S2). The lowest *δ*^13^C values were from subsection 1 (mode among individuals) or 2 (mean among individuals), and the highest was from subsection 6 (mode) or 8 (mean) (Table 1, Table 2 and Table S2). The main increase of *δ*^15^N values was from subsection 2 to 7 with a mean of 0.89‰ per subsection and decreased to a mean of 0.08‰ per subsection afterward (Figure 3, Table 2 and Table S2). The lowest *δ*^15^N values were from subsection 1 (mode) or 2 (mean), and the highest was from subsection 10 (both mode and mean) (Table 1, Table 2 and Table S2).

Values of *δ*^13^C of subsection 1 were significantly lower than subsections 6+ (Skillings–Mack test _SM_ = 57.13, *d. f.* = 10, *p* < 0.0001) (Table 3). Similarly, values of *δ*^15^N (_SM_ = 96.42, *d. f.* = 10, *p* < 0.0001) showed significant differences among subsections: subsections 1–3 were significantly lower than subsections 7+, and values of subsections 4–5 were significantly lower than subsections 10+ (Table 3).

A significant positive correlation between *δ*^13^C and *δ*^15^N values existed in six of the seven individuals we were able to check (Table S6). A significant positive correlation between *δ*^13^C and *δ*^15^N values also existed at the population level (Table S6).

### 3.2. Variation of Stable Isotopic Trajectories: Among Individuals and between Sexes

The specialization index of *δ*^15^N suggested that the studied *G. fabricii* were all generalists with low variability among the individuals (Table 1). Significant differences were present between the sexes: *χ*^2^ = 17.56, *d. f.* = 5, *p* < 0.0001. The specialization index of *δ*^13^C suggested that some of the studied individuals of *G. fabricii* were specialists in habitat usage, with high variability among the individuals (Table 1). The specialization index of *δ*^13^C was lower than 0.5 in three of six females and in one of eight males, and the latter was the lowest among the studied squid (*s* = 0.21). Significant differences were present between the sexes: *χ*^2^ = 92.21, *d. f.* = 5, *p* < 0.0001.

Both sexes reached the highest values of *δ*^15^N (and thus TL) in subsection 10. However, females reached the highest values of *δ*^13^C at subsection 9 (larger ML), while males at subsection 8 (smaller ML). Within-individual ontogenetic increase was smaller than within-population for both sexes. No significant differences were found between the sexes for within-individual increase: *n* = 13, *U* = 17.5, *p* = 0.67 smallest to largest and *n* = 13, *U* = 20.0, *p* = 0.95 first to last in *δ*^13^C; and *n* = 14, *U* = 23.0, *p* = 0.95 smallest to largest and *n* = 14, *U* = 22.0, *p* = 0.85 first to last in *δ*^15^N.

### 3.3. Isotopic Niches and Diet Models as a Basis of Life History Reconstruction

**Figure 4:**
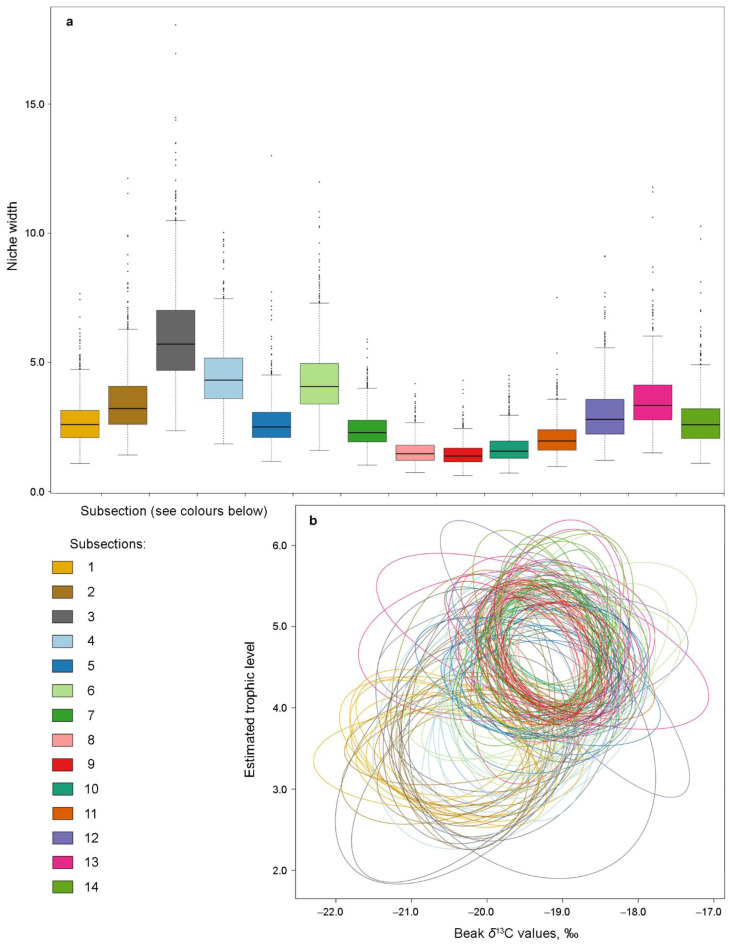
Isotopic niche width (**a**) and plot of ten random elliptical projections of niche region for each subsection (**b**) among the upper beak subsections of *Gonatus fabricii*, estimated in nicheROVER 1.1.0.

No clear pattern of ontogenetic changes in niche width was found when analysed by subsections. The widest niches were from subsections 3, 4, and 6, and the first and the last analysed subsections were of roughly equal width (Figure 4a,b, Table S7). Niches of subsections 1 and 2 had the least overlap with the rest (Figure 4b and Figure S2, Table 4).

**Figure 5:**
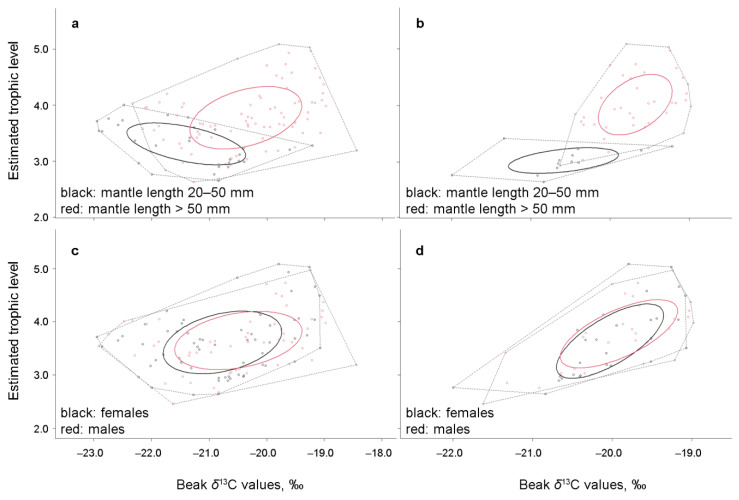
Isotopic niches in *Gonatus fabricii* at the population level, estimated in SIBER 2.1.6: (**a**) ontogenetic approach, all samples (West and East Greenland, and the Barents Sea), (**b**) ontogenetic approach, West Greenland, (**c**) Sexes, all samples (West and East Greenland, and the Barents Sea), and (**d**) sexes, West Greenland.

Based on nicheROVER, SIA values, and diet modelling analyses, population niches with SIBER should be compared among squid with ML < 20 mm (see Material and Methods), ML 20–50 mm, and ML > 50 mm. The niche of squid with ML > 50 mm was significantly wider than of squid with ML 20–50 mm, with low overlap between the niches (Figure 5a, Table 5). No significant differences were found between the isotopic niche width of males and females (Figure 5c,d, Table 5). These niches had significant overlap for both size classes (Figure 5c,d, Table 5 and Table S8). The pattern of ontogenetic niche increase and overlap in females and males was similar (when the sexes were analysed separately) (Table S8).

**Figure 6:**
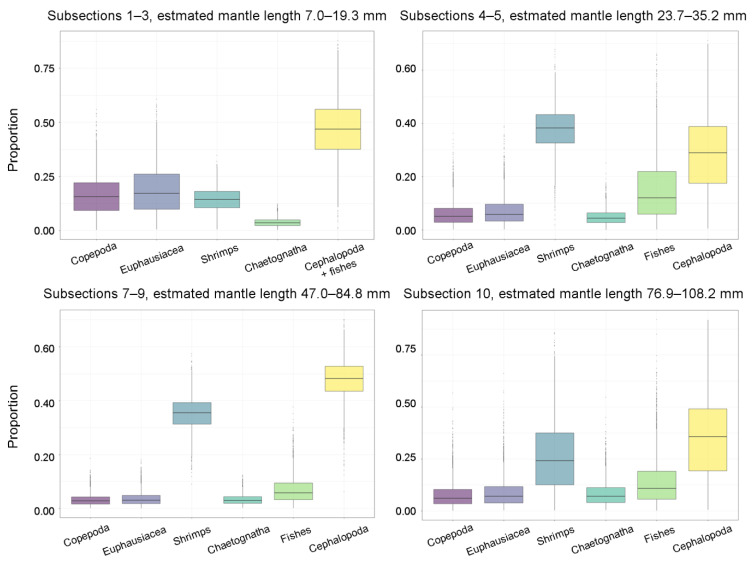
Relative contribution of prey to the diet (mean, box 25% and 75% percentiles, whiskers 5%, and 95% percentiles) in different upper beak subsections of *Gonatus fabricii* (as a proxy of different sizes) predicted by Bayesian mixing model SIMMR 0.4.5.

The diet corresponding to beak subsections 1–3, 4–5, 7–9, and 10 was modelled and compared. Chaetognaths were always a minor component of *G. fabricii*’s predicted diet (Figure 6, Table 6). Crustaceans constituted a half or more in subsections 1–3 and 4–5, or slightly less than a half in subsections 7–9 and 10 (Figure 6, Table 6). Combined, cephalopods, and fish formed a complementary pattern to crustaceans, i.e., they constituted slightly less than a half in subsections 1–3 and 4–5, and slightly more than a half in subsections 7–9 and 10 (Figure 6, Table 6). No significant differences in the predicted relative contribution of prey taxa (i.e., chaetognaths, crustaceans, and cephalopods + fish) were found among different subsections (Table S9): the described patterns are trends only. Cannibalism is predicted to be very important in the diet of *G. fabricii* (Figure 6, Table 6). Within crustaceans, the smallest analysed squid (subsections 1–3) were predicted to consume copepods, euphausiids, and shrimps in roughly equal ratios, which significantly differed from other size groups of *G. fabricii* (Table S9) who mostly relied on larger crustaceans, i.e., euphausiids and shrimps, than copepods (Figure 6, Table 6).

### 3.4. Beak Equations

The equations for ML and UCL were described as: ML = 3.25 × UCL^1.40^ (*n* = 86, *R*^2^ = 0.95, *p* < 0.0001) in West Greenland, and ML = 3.77 × UCL^1.34^ (*n* = 142, *R*^2^ = 0.93, *p* < 0.0001) overall in the Arctic (West and East Greenland, and the Barents Sea). The difference between real and predicted ML was 4.2 ± 2.44 mm for the West Greenland equation and 3.3 ± 1.72 mm for the overall Arctic equation. Updated URL equations (Table S3) outperformed previous ones cf. [27]: estimated ML was 5.1 ± 2.02 mm smaller than real ML vs. 12.2 ± 2.36 mm previously for the overall Arctic equation, and 6.1 ± 2.92 mm vs. 16.5 ± 3.51 mm, respectively, for the West Greenland equation.

## 4. Discussion

### 4.1. Ontogenetic Change in Isotopic Values, and Its Ecological Applications

Significant ontogenetic increase of both carbon and nitrogen isotopic values (and thus TL) in *G. fabricii* is now confirmed at the individual level. Previous SIA studies of *G. fabricii* [27,74], performed on whole beaks [27] or pieces of soft tissues [77] of multiple individuals (population analysis, not individual analysis), established this significant ontogenetic increase in populations. However, they did not find the causes for this increase and did not show at which sizes the main increase happens and to what degree changes in an individual follow that of a population. The major issue arising from this is that we need to apply a combination of individual and population SIA to study a species’ ecology more completely and from different perspectives (see practical recommendations for such an approach below).

The main ontogenetic increase in *δ*^13^C values takes place in squid from ML 7.0–7.4 mm to ML 30.9–44.2 mm. The increase in *δ*^13^C values is larger than expected based on dietary changes, i.e., mean 1.4‰ vs. about 1.0‰ [89]. The main ontogenetic increase in *δ*^15^N values (and TL) takes place in squid from ML 7.0–7.4 mm to ML 47.0–53.8 mm. The increase spans a mean of 1.5 TLs among studied individuals. Shifts in food spectra are the reason for changes in *δ*^15^N values and TL (as also highlighted by the diet models, below). However, other reasons such as individual movements may partly explain changes in *δ*^13^C values. Three types of migrations are known for *G. fabricii*. The first involves horizontal migration, the second is ontogenetic downward migration, and the third is diurnal vertical migration [27,31,32,34,36,37,38,39,40,41,42,113]. There is an occasional presence of squid with ML 20–50 mm deeper than 200 m [36,38,113,114,115,116], while they are supposed to exclusively inhabit epipelagic layers [42], which means the migrations outlined above also have a role in ontogenetic increase of *δ*^13^C values. It is clear that squid with ML 30.9–44.2 mm can occasionally exploit the whole water column when we combine these literature data on depth distribution with *δ*^13^C values reported here and in Golikov et al. [27].

The ML values highlighted by *δ*^15^N and TL analyses are used to group the squid and assess the species’ assimilated diet. Reported results on diet are generally in accordance with previous studies using stomach contents [36,43,44,45,46,115,116] that showed crustaceans are major prey for *G. fabricii* with the increasing importance of fish and cephalopods in individuals larger than ML 54–70 mm [44,45,46]. However, assimilated diet predicted by SIA data often does not entirely coincide with stomach contents analysis results in cephalopods [28,30,117]. Moreover, food spectra changes in *G. fabricii* were expected to happen due to the hook appearance at its arms and tentacular clubs [43,44,45,46], which exactly fits the predictions from this study. *Gonatus fabricii* already reaches the highest TL (adult diet) at ML 76.9–108.2 mm, with the maximum ML recorded for *G. fabricii* being 389 mm [46]. Such behavior is also detected in *Berryteuthis magister* (Berry, 1913) [118], a gonatid squid from the North Pacific that does not significantly change the size of the fish it preys upon throughout ontogenesis [48]. *Dosidicus gigas* (d’Orbigny [in 1834–1847], 1835) [119], a large squid from the Pacific, is also known to rely on small prey, despite that it is occasionally hunting for large prey [120,121]. Cannibalism is well-known for *G. fabricii* [36,44,46,115] and other gonatids [48,122] and the predicted diet models from this study underline its importance. Previous studies on stomach contents did not estimate prey sizes of *G. fabricii*, but the Pacific gonatids are known to eat fish and squids of 15–150% of their ML [48,122].

### 4.2. Variation of Stable Isotopic Trajectories: Among Individuals and between Sexes

Substantial variation among individual ontogenetic stable isotopic trajectories is found. We applied the specialization index *s* to explore it further. This is the first application of *s* to stable isotopic data from marine invertebrates to the authors’ knowledge. Specialization index *s* of *δ*^15^N shows that *G. fabricii* is a generalist predator, as most predatory squids are e.g., [48,50,120,121,122,123]. Significant differences in *s* of *δ*^15^N between the sexes in *G. fabricii* are influenced by only one individual: *s* values were 0.95–0.98 in all individuals, except female F5 with *s* = 0.83. Specialization index *s* of *δ*^13^C shows that *G. fabricii* is a specialist in habitat usage. In turn, it suggests variability in migrations among individuals. Females have much higher variation in habitat usage than males, resulting in lower specialization index *s* in *δ*^13^C, and females reach the highest *δ*^13^C values at larger sizes than males. Thus, at least part of the variation in *δ*^13^C is explained by these differences between the sexes. The greater migratory capability of females is known in oceanic squids *Ommastrephes bartramii* (Lesueur, 1821) [49,124] and *D. gigas* [123]. The niche width in *G. fabricii* is similar in females and males, with significant niche overlap between the sexes. This means that high individual variation in habitat usage and different migratory capabilities between sexes are not enough to differentiate niches between females and males due to individual squid’s highly generalist diets. These results contrast with previous studies on other cephalopods where significant differences in niche width were found between sexes [30,49,123,125].

### 4.3. Life Cycle of Gonatus fabricii

Previous attempts to differentiate between ecological roles during the ontogenesis of *G. fabricii* mostly used maturity stages (Table 7). The maturity stages, however, are not a good proxy for an ecological role as there is a strong overlap in ML of squid in different maturity stages (Table 7). An overview of ontogenetic changes in body shape, mass, and reproductive indices [36,39,40,42] suggests a rather slow pace of changes in ecological role, perhaps due to slow growth. However, these characters alone cannot be relied upon, as shown above. A previous study that applied SIA in *G. fabricii* [27] showed that the changes in the ecological role might happen faster than previously supposed [42]. Clearly separate ecological roles, which *G fabricii* occupies during ontogenesis, are established in this study (Table 7). This is achieved by a combination of individual and population SIA. The life history of *G. fabricii* is as follows: (1) epipelagic squid with ML < 20 mm prey mostly on crustaceans, especially copepods (as per stomach contents analysis) or copepods and larger crustaceans in equal proportions (as per SIA prediction); (2) squid with ML 20–50 mm mostly stay in the epipelagic layer, but occasionally exploit the whole water column, with their food spectra shifting to larger Crustacea, fish, and cephalopods; (3) squid with ML > 50 mm mostly prey on fish and cephalopods with a high proportion of cannibalism, and stay in the meso- and bathypelagic layers (occasionally might be recorded in the epipelagic layers, as they have daily vertical migrations); and 4) non-feeding and almost non-moving bathypelagic gelatinous females with ML > 200 mm (Table 7).

### 4.4. Ecosystem Implications

Dividing the life history of *G. fabricii* into distinct ecological roles provides baseline data on cephalopod biology and in Arctic marine ecosystem studies. Active cephalopod predators are often compared with bony fish in terms of ecological role. However, the life histories of these taxa are very different [130,131,132]. When based on a sufficient number of beak subsections, individual life history SIA reconstructions of cephalopods [47,52,66] strongly suggest that cephalopods change their ecological roles earlier in their ontogenesis compared to fish cf. [133,134,135]. The growth speed of juvenile *G. fabricii* in West Greenland was estimated to be 0.13–0.18 mm day^−1^ [136] and 0.27 mm day^−1^ [38]. This suggests that squid remains in a ‘pure’ epipelagic stage (ML < 20 mm) for 2.5–4.2 months, then transition their food spectra and habitat (ML 20–50 mm) for 3.7–6.3 months, with overall life expectancy in West Greenland of up to three years [36]. Thus, longer-living cold-water Arctic squid, which spend much of their life in the relatively stable deep-sea environment, still have major ecological changes during early ontogenesis, cf. the large Antarctic squid and octopus [51,52,66].

A high TL of 5.0 or above is only known in polar squids from the Arctic [27], and this study and Antarctic [82,137,138]. Very high *δ*^15^N values in the beaks of *Histioteuthis atlantica* (Hoyle 1885) [139] from Subantarctic waters suggest comparably high TL, as other squids in the area showed much lower *δ*^15^N values in their beaks [140]. The high TL of *G. antarcticus* Lönnberg, 1898 [141] is most likely explained in a similar way as *G. fabricii* (see above).

### 4.5. Modelling and Analyses Implications and Recommendations

Previous studies used eye lenses and gladii of cephalopods to analyse individual ontogenetic stable isotopic trajectories; however, they failed to identify the fast pace of changes in cephalopod ecological role early in ontogenesis [48,49,50,123,142,143,144,145,146,147,148]. Unlike beaks, neither eye lenses nor gladii were proven to have daily growth increment deposition [62,63,64,65]. Considering beak utility when necessary methodological considerations were performed for the anterior-most and posterior-most parts [51,61,62,63,69], beaks are recommended for ontogenetic SIA studies in the future. Analysis of individual ontogenetic stable isotopic trajectories from beak crests provides precise sizes of ecological role changes, especially if coupled with analysis of multiple individuals with their whole beaks powdered. The recommendation is to use one hundred beaks in equal proportions of the small, medium, and large sizes for population SIA, which is a large enough sample size [27,76]. With each size group being *n* > 30, it would not bias the SIBER niche analysis outcome [149]. At least ten large beaks are recommended for individual ontogenetic SIA, taking consecutive pieces along the crest. Combining these approaches would give an extensive overview of the species’ life history, together with its reconstruction with exact sizes, if a beak equation to estimate squid size exists for the study species. Equations to estimate ML of *G. fabricii* from URL and UHL exist for the Barents Sea, West Greenland, East Greenland, and the overall Arctic [27], and this study, and equations to estimate squid ML from UCL only exist for West Greenland and the overall Arctic (this study). Our recommendation is to use the equation for the specific area of the Arctic (i.e., the Barents Sea, West Greenland, or East Greenland) if it is known where the beaks come from, and to use the overall Arctic equation in the absence of such information.

For future Arctic ecosystem modelling we recommend using the size-specific ecological role periodization established here. The ecological role of *G. fabricii* is misrepresented in current models. The dataset of Planque et al. [13] is used as a basis for most of the Barents Sea ecosystem models [14,15,17,18,19,20]. In these, *G. fabricii* only has 12 prey taxa (all Crustacea and *G. fabricii* itself, i.e., no fish) and 23 predators, cf. at least 49 and 47, respectively [27]. Thus, these models completely miss the role of *G. fabricii* as a fish consumer. The older models [10,11,12] and those reusing them as a baseline [16] also do not reflect the ecological role of *G. fabricii*. Dommasnes et al. [10], Blanchard et al. [12], and Murphy et al. [16] are missing some groups of prey and predators: in particular, they miss cannibalism in *G. fabricii*. Moreover, they extrapolate biomass values of *G. fabricii* from the Nordic Seas to the Barents Sea [10,12,16], which has been already shown to be incorrect elsewhere [34]. The Nordic Basin and the Barents Sea are very different in their oceanographic conditions, and thus in their role in the range of *G. fabricii* [34,37]. For West Greenland, Pedersen and Zeller [11] provided a better description of the trophic role of *G. fabricii*, however, suffer from biomass estimation and P/B and Q/B coefficients for the species.

The only *G. fabricii* biomass assessment where large individuals were included was performed by Bjørke and Gjøsaeter [31] for the Nordic Seas, which largely covers the Norwegian Sea. It shows 1.5 million t of squid in the upper 30 m layer and 8 million t of meso- and bathypelagic squid [31]. Applying the ecological stages established here might prove difficult, as: (1) it is unknown how much ‘epipelagic’ squid are with ML < 20 mm and how much belong to other groups, and to which groups; (2) the layer between the upper 30 m and mesopelagic layer is missing; (3) it is unknown how much of meso- and bathypelagic squid are with ML < 50 mm and ML > 50 mm; and 4) the proportion of gelatinous females in the latter group is also unknown. Extrapolation based on Figure 3 from Bjørke and Gjøsaeter [31] suggests the whole estimated ‘epipelagic’ of 1.5 million t are squid with ML < 20 mm, then there are 2.5 million t of squid with ML of 20–50 mm. If the sex ratio is assumed to be equal (it has never been assessed), and surviving to spawn is 5% [31], then the biomass of gelatinous females would be 0.25 million t. The biomass proportion of *G. fabricii* with a different ecology in the Nordic Seas would be approximately 12% squid with ML < 20 mm, 21% squid with ML 20–50 mm, 67% squid with ML > 50 mm, and 0.02% gelatinous females. However, *G. fabricii* is supposed to live up to two years in the Nordic Seas [150], and this is the assumption Bjørke and Gjøsaeter [31] relied on. In this study, *G. fabricii* from West Greenland is assessed to define the ontogenetic stages with different ecology, which is supposed to live up to three years [36].

## 5. Conclusions

We detect a significant ontogenetic increase of *δ*^13^C and *δ*^15^N values (and TL) in *G. fabricii* in individual squid. There is substantial variation among stable isotope trajectories in individual *G. fabricii* beaks, with *δ*^13^C values having higher variation among individuals, especially in females, than *δ*^15^N values. At the same time, isotopic niches are not different between the sexes, and are highly overlapping. Exact sizes when the species occupies different ecological roles, both ML and mass, are found. We recommend that this ecological periodization (with four clearly distinct stages) be used in future Arctic marine ecosystem modelling. Currently, all existing Arctic ecosystem models fail to reflect the ecological role of *G. fabricii* correctly, despite its immense importance in Arctic ecosystems. From supposedly three years of squid ontogenesis in West Greenland cf. [36], stage 1 is expected to last for 2.5–4.2 months followed by 3.7–6.3 months stage 2. Thus, the main changes in Arctic squid ecology occur during the first 17–29% of the supposed life cycle and are coupled with a fast size increase. We find that beaks have a superior capacity to visualize ontogenetic changes in squid ecology compared to other hard structures such as eye lenses and gladii. To assess the variation in stable isotope data, the specialization index *s* is successfully applied for the first time in cephalopods. Finally, an equation to estimate the ML of *G. fabricii* from UCL is provided, and existing equations to estimate ML and mass from URL are updated.

## Figures and Tables

**Table 1:** Values of *δ*^13^C and *δ*^15^N, estimated trophic level, and specialization index of all analysed individuals of *Gonatus fabricii* (*n* = 14), and mean among the individuals, where applicable. ML: mantle length, TL: estimated trophic level, *s*: specialization index, *n*: sample size, n/a: not analysed, ---: not applicable.

Squid	Sex	ML, mm	Character	*n* Successfully Analysed/ *n* Available	Minimum—Maximum (Mean ± SE)	*s*	Minimum Value, Subsection Number	Maximum Value, Subsection Number	Range of Values	
									Minimum to Maximum	Newest to Oldest
F1	Female	214	*δ*^13^C, ‰	13/13	−20.5 to −18.6 (−19.2 ± 0.19)	0.78	1	7	1.87	1.82
			*δ*^15^N, ‰	13/13	7.9–13.5 (11.8 ± 0.54)	0.97	2	10	4.60	4.08
			TL	13/13	3.0–4.5 (4.0 ± 0.14)	---	2	10	1.47	1.07
F2	Female	215	*δ*^13^C, ‰	13/13	−20.5 to −19.4 (−19.9 ± 0.09)	0.45	3	8	1.04	0.71
			*δ*^15^N, ‰	13/13	8.6–13.5 (11.4 ± 0.49)	0.96	1	8	4.90	3.58
			TL	13/13	3.2–4.5 (3.9 ± 0.13)	---	1	8	1.29	0.94
F3	Female	238	*δ*^13^C, ‰	11/15	−20.5 to −18.9 (−19.6 ± 0.17)	0.71	1	14	1.60	1.42
			*δ*^15^N, ‰	12/15	10.3–16.7 (13.9 ± 0.63)	0.97	6	13	6.42	4.30
			TL	12/15	3.6–5.3 (4.6 ± 0.17)	---	6	13	1.69	1.13
F4	Female	249	*δ*^13^C, ‰	6/14	−19.4 to −18.6 (−19.1 ± 0.12)	0.40	4	5	0.75	0.06
			*δ*^15^N, ‰	10/14	6.4–15.5 (11.9 ± 0.82)	0.98	2	4	9.09	8.85
			TL	10/14	3.0–5.4 (4.5 ± 0.22)	---	2	4	2.39	2.33
F5	Female	257	*δ*^13^C, ‰	9/13	−19.8 to −19.0 (−19.3 ± 0.09)	0.37	4	6	0.76	0.61
			*δ*^15^N, ‰	10/13	13.7–16.3 (15.6 ± 0.25)	0.83	4	11	2.62	1.61
			TL	10/13	4.5–5.2 (5.0 ± 0.07)	---	4	11	0.69	0.42
F6 ^1^	Female	230	*δ*^13^C, ‰	15/15	−20.2 to −18.5 (−19.2 ± 0.11)	0.57	1	15	1.71	1.71
			*δ*^15^N, ‰	15/15	11.2–16.7 (15.0 ± 0.45)	0.96	1	15	5.58	5.58
			TL	15/15	3.6–5.1 (4.7 ± 0.12)	---	1	15	1.47	1.47
M1	Male	216	*δ*^13^C, ‰	9/12	−20.1 to −18.5 (−19.0 ± 0.17)	0.66	1	4	1.60	1.05
			*δ*^15^N, ‰	9/12	8.9–14.4 (12.5 ± 0.65)	0.97	1	10	5.41	4.52
			TL	9/12	3.3–4.7 (4.2 ± 0.17)	---	1	10	1.42	1.19
M2	Male	221	*δ*^13^C, ‰	11/11	−21.2 to −19.3 (−19.9 ± 0.20)	0.77	1	5	1.90	1.59
			*δ*^15^N, ‰	11/11	6.1–12.4 (9.9 ± 0.75)	0.98	1	10	6.23	5.86
			TL	11/11	3.0–4.6 (4.0 ± 0.20)	---	1	10	1.64	1.54
M3	Male	269	*δ*^13^C, ‰	14/14	−21.0 to −19.4 (−20.0 ± 0.15)	0.70	1	8	1.57	1.20
			*δ*^15^N, ‰	14/14	9.5–15.4 (13.1 ± 0.57)	0.97	2	10	5.94	4.29
			TL	14/14	3.8–5.3 (4.7 ± 0.15)	---	2	10	1.56	1.13
M4	Male	285	*δ*^13^C, ‰	15/16	−20.3 to −18.2 (−19.0 ± 0.16)	0.74	1	12	2.18	1.02
			*δ*^15^N, ‰	15/16	5.6–11.5 (9.6 ± 0.53)	0.97	1	11	5.97	4.51
			TL	15/16	3.0–4.6 (4.1 ± 0.14)	---	1	11	1.57	1.19
M5	Male	252	*δ*^13^C, ‰	14/14	−19.6 to −18.9 (−19.2 ± 0.05)	0.21	3	6	0.72	0.11
			*δ*^15^N, ‰	14/14	6.4–12.3 (10.3 ± 0.49)	0.96	3	10	5.95	1.75
			TL	14/14	3.0–4.6 (4.1 ± 0.13)	---	3	10	1.56	0.46
M6	Male	284	*δ*^13^C, ‰	15/15	−20.8 to −19.2 (−19.7 ± 0.12)	0.62	1	14	1.55	1.11
			*δ*^15^N, ‰	15/15	11.1–16.6 (14.5 ± 0.43)	0.96	1	13	5.49	3.18
			TL	15/15	3.8–5.3 (4.7 ± 0.11)	---	1	13	1.45	0.84
M7	Male	298	*δ*^13^C, ‰	4/17 ^2^	−19.5 to −19.1 (−19.2 ± 0.08)	n/a ^2^	n/a ^2^	n/a ^2^	n/a ^2^	n/a ^2^
			*δ*^15^N, ‰	13/17	10.3–15.4 (13.8 ± 0.47)	0.96	3	9	5.09	4.27
			TL	13/17	4.1–5.4 (5.0 ± 0.12)	---	3	9	1.34	1.12
M8	Male	325	*δ*^13^C, ‰	12/17	−20.7 to −19.3 (−19.7 ± 0.11)	0.51	1	6	1.43	0.80
			*δ*^15^N, ‰	14/17	10.7–15.1 (13.5 ± 0.42)	0.95	2	7	4.41	2.67
			TL	14/17	3.7–4.9 (4.5 ± 0.11)	---	2	7	1.16	0.70
Mean ± SE	---	---	*δ*^13^C, ‰	13 individuals (all Except M7)	n/a	0.58 ± 0.05	Mode 1, 1.77 ± 0.34	Mode 6, 8.46 ± 1.08	0.7–2.2 (1.4 ± 0.13)	0.1–1.8 (1.0 ± 0.15)
			*δ*^15^N, ‰	14 individuals	n/a	0.96 ± 0.01	Mode 1, 2.14 ± 0.39	Mode 10, 10.07 ± 0.72	2.6–9.1 (5.6 ± 0.37)	1.6–8.9 (4.2 ± 0.49)
			TL	14 individuals	n/a	---	Mode 1, 2.14 ± 0.39	Mode 10, 10.07 ± 0.72	0.7–2.4 (1.5 ± 0.10)	0.4–2.3 (1.1 ± 0.13)

^1^ gelatinous degeneration; ^2^ only subsections 4, 7, 15, and 17 were successfully analysed for *δ*^13^C values, thus, this individual was excluded from some analyses.

**Table 2:** Mean values of *δ*^13^C and *δ*^15^N, and estimated trophic level of all analysed upper beak subsections of *Gonatus fabricii* (*n* = 179). ML: mantle length, TL: estimated trophic level, *n*: sample size, n/a: no results, ---: not applicable.

Subsection Number	Character	*n* Successfully Analysed/ n Available	Minimum—Maximum (Mean ± SE)	Subsection Number	*n* Successfully Analysed/ n Available	Minimum—Maximum (Mean ± SE)
1	*δ*^13^C, ‰	11/14	−21.2 to −19.3 (−20.5 ± 0.15)	10	10/14	−19.7 to −18.7 (−19.2 ± 0.12)
	*δ*^15^N, ‰	11/14	5.6–11.2 (9.1 ± 0.57)		12/14	11.2–15.8 (14.2 ± 0.46)
	TL	11/14	3.0–3.8 (3.5 ± 0.10)		12/14	4.4–5.3 (4.8 ± 0.09)
	Est. ML, mm	14/14	7.0–7.4 (7.3 ± 0.04)		14/14	76.9–108.2 (86.9 ± 2.49)
	Est. mass, g	14/14	0.03–0.04 (0.033 ± 0.0005)		14/14	15.2–36.7 (20.8 ± 1.8)
2	*δ*^13^C, ‰	9/14	−20.9 to −19.3 (−20.2 ± 0.17)	11	11/14	−19.7 to −18.5 (−19.2 ± 0.12)
	*δ*^15^N, ‰	12/14	5.7–11.9 (9.3 ± 0.66)		13/14	11.1–16.3 (13.9 ± 0.49)
	TL	12/14	3.0–4.2 (3.6 ± 0.13)		13/14	4.3–5.2 (4.8 ± 0.09)
	Est. ML, mm	14/14	11.6–12.8 (12.3 ± 0.10)		14/14	92.4–133.3 (106.5 ± 3.31)
	Est. mass, g	14/14	0.11–0.14 (0.13 ± 0.003)		14/14	24.4–63.0 (35.4 ± 3.2)
3	*δ*^13^C, ‰	11/14	−21.0 to −18.9 (−19.9 ± 0.18)	12	9/13	−19.7 to −18.2 (−19.1 ± 0.16)
	*δ*^15^N, ‰	13/14	6.4–13.9 (10.2 ± 0.70)		10/13	11.2–16.5 (13.7 ± 0.65)
	TL	13/14	3.0–5.0 (3.8 ± 0.16)		10/13	4.3–5.2 (4.7 ± 0.12)
	Est. ML, mm	14/14	17.3–19.3 (18.5 ± 0.17)		13/13	113.5–159.8 (129.5 ± 3.73)
	Est. mass, g	14/14	0.3–0.4 (0.37 ± 0.01)		13/13	41.6–100.7 (58.3 ± 4.8)
4	*δ*^13^C, ‰	13/14	−20.2 to −18.5 (−19.6 ± 0.13)	13	10/12	−20.0 to −18.3 (−19.2 ± 0.15)
	*δ*^15^N, ‰	13/14	8.2–15.5 (11.7 ± 0.61)		12/12	10.7–16.7 (13.9 ± 0.65)
	TL	13/14	3.4–5.4 (4.1 ± 0.15)		12/12	4.1–5.3 (4.8 ± 0.13)
	Est. ML, mm	14/14	23.7–26.8 (25.5 ± 0.25)		12/12	135.9–162.0 (148.6 ± 2.67)
	Est. mass, g	14/14	1.4–1.9 (1.7 ± 0.05)		12/12	66.2–100.7 (81.3 ± 3.7)
5	*δ*^13^C, ‰	12/14	−20.1 to −18.6 (−19.3 ± 0.14)	14	8/9	−19.8 to −18.6 (−19.2 ± 0.13)
	*δ*^15^N, ‰	14/14	8.2–15.1 (12.4 ± 0.50)		9/9	10.1–16.4 (14.3 ± 0.78)
	TL	14/14	3.7–4.9 (4.4 ± 0.09)		9/9	4.0–5.4 (4.9 ± 0.15)
	Est. ML, mm	14/14	30.9–35.2 (33.3 ± 0.35)		9/9	159.4–190.3 (173.5 ± 3.09)
	Est. mass, g	14/14	1.4–1.9 (1.7 ± 0.05)		9/9	100.1–152.8 (121.3 ± 5.4)
6	*δ*^13^C, ‰	11/14	−20.2 to −18.5 (−19.3 ± 0.16)	15	6/6	−19.7 to −18.5 (−19.2 ± 0.17)
	*δ*^15^N, ‰	12/14	9.9–15.7 (12.5 ± 0.61)		6/6	10.5–16.7 (14.4 ± 0.85)
	TL	12/14	3.6–5.0 (4.4± 0.14)		6/6	4.3–5.3 (4.8 ± 0.14)
	Est. ML, mm	14/14	38.7–44.2 (41.8 ± 0.45)		6/6	184.0–219.9 (199.8 ± 4.90)
	Est. mass, g	14/14	2.4–3.5 (3.1 ± 0.1)		6/6	145.0–222.0 (176.1 ± 10.3)
7	*δ*^13^C, ‰	13/14	−20.0 to −18.6 (−19.2 ± 0.10)	16	2/3	−19.8 to −19.3 (−19.6 ± 0.23)
	*δ*^15^N, ‰	13/14	10.7–16.2 (13.7 ± 0.49)		2/3	10.1–14.2 (12.1 ± 2.06)
	TL	13/14	4.0–5.4 (4.7 ± 0.10)		2/3	4.2–4.7 (4.4 ± 0.23)
	Est. ML, mm	14/14	47.0–53.8 (50.9 ± 0.56)		3/3	209.5–216.6 (211.9 ± 2.34)
	Est. mass, g	14/14	4.0–5.8 (5.1 ± 0.2)		3/3	203.1–221.2 (209.1 ± 6.0)
8	*δ*^13^C, ‰	11/14	−19.7 to −18.8 (−19.3 ± 0.08)	17	2/2	−19.9 to −19.5 (−19.7 ± 0.24)
	*δ*^15^N, ‰	12/14	10.7–16.1 (13.6 ± 0.57)		2/2	13.5–15.3 (14.4 ± 0.88)
	TL	12/14	4.2–5.2 (4.7 ± 0.11)		2/2	4.5–5.4 (4.9 ± 0.44)
	Est. ML, mm	14/14	55.8–64.0 (60.5 ± 0.67)		2/2	236.1–244.1 (238.7 ± 2.69)
	Est. mass, g	14/14	6.3–9.1 (8.0 ± 0.2)		2/2	276.5–301.6 (284.8 ± 8.4)
9	*δ*^13^C, ‰	12/14	−19.7 to −18.8 (−19.2 ± 0.09)			
	*δ*^15^N, ‰	13/14	10.7–16.1 (13.8 ± 0.50)			
	TL	13/14	4.3–5.4 (4.7 ± 0.10)			
	Est. ML, mm	14/14	65.0–84.8 (72.1 ± 1.50)			
	Est. mass, g	14/14	9.3–19.5 (12.7 ± 0.8)			

**Table 3:** Comparison of (a) *δ*^13^C and (b) *δ*^15^N values, and (c) estimated trophic level among different upper beak subsections in *Gonatus fabricii*, using the Skillings–Mack test with the Nemenyi post hoc test. Significant *p*-values are in **bold**. TL: estimated trophic level, ---: not applicable.

(a) *δ*^13^C values: _SM_ = 57.13, *d. f.* = 10, ***p* < 0.0001**										
Subsections	1 (anterior)	2	3	4	5	6	7	8	9	10 (posterior)
2	0.88	---	---	---	---	---	---	---	---	---
3	0.93	1.00	---	---	---	---	---	---	---	---
4	0.68	1.00	1.00	---	---	---	---	---	---	---
5	0.0540	0.88	0.83	0.98	---	---	---	---	---	---
6	**0.0011**	0.22	0.17	0.43	0.99	---	---	---	---	---
7	**0.0017**	0.28	0.22	0.51	1.00	1.00	---	---	---	---
8	**0.0046**	0.43	0.35	0.68	1.00	1.00	1.00	---	---	---
9	**0.0375**	0.83	0.76	0.96	0.99	1.00	1.00	1.00	---	---
10	**0.0008**	0.19	0.14	0.39	0.99	1.00	1.00	1.00	1.00	---
11	**0.0090**	0.56	0.47	0.80	1.00	1.00	1.00	1.00	1.00	1.00
(b) *δ*^15^N values: _SM_ = 96.42, *d. f.* = 10, ***p* < 0.0001**										
Subsections	1 (anterior)	2	3	4	5	6	7	8	9	10 (posterior)
2	1.00	---	---	---	---	---	---	---	---	---
3	1.00	1.00	---	---	---	---	---	---	---	---
4	1.00	1.00	1.00	---	---	---	---	---	---	---
5	0.98	0.94	1.00	1.00	---	---	---	---	---	---
6	0.61	0.49	0.77	0.98	1.00	---	---	---	---	---
7	**0.0179**	**0.0097**	**0.0382**	0.21	0.44	0.93	---	---	---	---
8	**0.0097**	**0.0051**	**0.0218**	0.14	0.34	0.86	1.00	---	---	---
9	**0.0041**	**0.0020**	**0.0097**	0.08	0.21	0.73	1.00	1.00	---	---
10	**<0.0001**	**<0.0001**	**0.0001**	**0.0020**	**0.0097**	0.12	0.94	0.98	1.00	---
11	**0.0020**	**0.0010**	**0.0051**	**0.0457**	0.14	0.61	1.00	1.00	1.00	1.00
(c) TL: _SM_ = 96.02, *d. f.* = 10, ***p* < 0.0001**										
Subsections	1 (anterior)	2	3	4	5	6	7	8	9	10 (posterior)
2	1.00	---	---	---	---	---	---	---	---	---
3	1.00	1.00	---	---	---	---	---	---	---	---
4	1.00	1.00	1.00	---	---	---	---	---	---	---
5	0.98	0.94	1.00	1.00	---	---	---	---	---	---
6	0.59	0.47	0.75	0.98	1.00	---	---	---	---	---
7	**0.0198**	**0.0108**	**0.0418**	0.22	0.47	0.94	---	---	---	---
8	**0.0088**	**0.0045**	**0.0198**	0.13	0.32	0.86	1.00	---	---	---
9	**0.0045**	**0.0023**	**0.0108**	0.08	0.22	0.77	1.00	1.00	---	---
10	**<0.0001**	**<0.0001**	**0.0001**	**0.0023**	**0.0108**	0.14	0.94	0.98	1.00	---
11	**0.0018**	**0.0009**	**0.0009**	**0.0418**	0.13	0.61	1.00	1.00	1.00	1.00

**Table 4:** Overlap among isotopic niches, estimated in nicheROVER 1.1.0 for different upper beak subsections of *Gonatus fabricii*. Significant overlap values are in **bold**. ---—not applicable.

Subsections														
	1	2	3	4	5	6	7	8	9	10	11	12	13	14
1	---	**94.54**	**95.57**	**73.17**	14.36	52.09	7.87	1.82	0.92	0.94	2.11	3.73	7.31	6.90
2	**87.76**	---	**96.40**	**81.53**	26.34	**63.11**	16.26	5.67	3.59	3.94	6.25	9.37	15.46	13.71
3	**64.17**	**77.70**	---	**85.38**	49.94	**74.85**	39.04	24.05	19.81	20.21	25.49	31.27	39.44	34.69
4	43.37	**64.36**	**94.27**	---	**76.01**	**91.31**	**67.94**	50.84	44.46	44.59	52.00	59.46	**68.66**	**62.35**
5	19.66	46.73	**93.95**	**95.75**	---	**96.74**	**86.75**	**75.14**	**71.31**	**70.19**	**77.19**	**85.22**	**91.07**	**82.15**
6	26.18	47.52	**90.00**	**93.44**	**82.84**	---	**79.56**	**64.64**	**60.00**	**60.81**	**67.85**	**75.89**	**82.54**	**75.06**
7	7.87	25.81	**86.85**	**95.25**	**84.34**	**96.73**	---	**85.45**	**82.75**	**85.86**	**90.57**	**94.27**	**96.71**	**93.63**
8	5.98	25.45	**91.40**	**97.89**	**90.07**	**98.01**	**97.18**	---	**92.65**	**92.56**	**95.01**	**97.42**	**98.63**	**96.93**
9	4.30	22.28	**91.84**	**97.81**	**91.94**	**98.01**	**96.85**	**94.05**	---	**94.42**	**95.90**	**98.21**	**98.89**	**96.42**
10	3.24	18.77	**88.58**	**96.41**	**87.66**	**97.13**	**96.01**	**90.77**	**91.08**	---	**95.69**	**97.80**	**98.72**	**95.62**
11	4.01	18.86	**86.16**	**94.95**	**84.23**	**96.46**	**94.72**	**87.02**	**86.57**	**90.21**	---	**96.46**	**98.03**	**94.24**
12	4.19	17.26	**79.42**	**89.10**	**78.23**	**92.32**	**87.87**	**77.41**	**77.18**	**81.84**	**86.45**	---	**94.47**	**87.57**
13	5.25	18.53	**77.89**	**87.88**	**74.19**	**90.86**	**86.68**	**74.64**	**73.32**	**78.17**	**83.96**	**90.20**	---	**86.94**
14	5.25	17.99	**77.38**	**89.41**	**70.07**	**90.98**	**90.02**	**79.12**	**75.21**	**79.82**	**85.71**	**90.71**	**94.22**	---

**Table 5:** Isotopic niche metrics (TA, SEA_c_, and SEA_b_) and overlap in *Gonatus fabricii* within different studied areas, estimated in SIBER 2.1.6. SEA_b_ values are means ± SD. Significant *p*-values are in **bold**. Significant overlap values are in **bold**. ML: mantle length, *n*: sample size, ---: not applicable.

Parameter	Arctic ^1^		West Greenland	
	ML 20–50 mm	ML > 50 mm	ML 20–50 mm	ML > 50 mm
*n*	29	85	13	40
TA	2.77	6.26	1.20	2.25
SEA_c_	1.04	1.55	0.47	0.71
SEA_b_	1.04 ± 0.20	1.55 ± 0.17	0.48 ± 0.15	0.71 ± 0.12
ML 20–50 mm (*p*-values)	---	**0.9688**	---	0.91
ML > 50 mm (*p*-values)	**0.0312**	---	0.09	---
Overlap, %	17.88	11.65	0.00	0.00
Parameter	Females	Males	Females	Males
*n*	62	56	28	29
TA	6.01	6.58	3.70	3.39
SEA_c_	1.78	1.79	1.06	1.12
SEA_b_	1.78 ± 0.23	1.79 ± 0.24	1.10 ± 0.22	1.16 ± 0.22
Females (*p*-values)	---	0.51	---	0.57
Males (*p*-values)	0.49	---	0.43	---
Overlap, %	**86.64**	**85.96**	**90.55**	**85.77**

^1^ West Greenland, East Greenland, and the Barents Sea.

**Table 6:** Relative contribution of prey to the diet in different upper beak subsections of *Gonatus fabricii*, predicted by Bayesian mixing model SIMMR 0.4.5. Relative contributions are mean ± SD. ML: mantle length, *n*: sample size, ---: not applicable.

Parameter	Subsections			
	1–3	4–5	7–9	10
Est. ML, mm	7.0–19.3	23.7–35.2	47.0–84.8	76.9–108.2
*n* ^1^	23	16	20	5
1. Crustacea, %	49.6 ± 25.2 ^2^	51.2 ± 18.1 ^2^	42.0 ± 10.8 ^2^	42.7 ± 29.9 ^2^
1.1. Copepoda, %	16.4 ± 8.9	6.1 ± 4.4	3.2 ± 2.1	7.8 ± 6.4
1.2. Euphausiacea, %	18.7 ± 11.0	7.3 ± 5.4	3.6 ± 2.5	8.9 ± 7.4
1.3 Shrimps, %	14.5 ± 5.3	37.8 ± 8.3	35.2 ± 6.2	26.0 ± 16.1
2. Chaetognatha, %	3.9 ± 2.0	4.9 ± 2.8	3.3 ± 1.9	8.3 ± 5.9
3. Cephalopoda + fish, %	46.5 ± 13.4 ^3^	43.9 ± 25.9 ^3^	54.7 ± 12.5 ^3^	49.0 ± 30.9 ^3^
3.1. Cephalopoda, %	23.3 ± 6.7 ^3^	28.6 ± 14.0 ^3^	47.8 ± 7.5 ^3^	34.7 ± 18.8 ^3^
3.2. Fish, %	23.2 ± 6.7 ^3^	15.3 ± 11.9 ^3^	6.9 ± 5.0 ^3^	14.3 ± 12.1 ^3^

^1^ Fitting only, see Materials and Methods for checking; ^2^ Crustacea estimated as three crustacean taxa combined; ^3^ Cephalopoda + fish estimated together for subsections 1–3, and separately for the rest (see Materials and Methods).

**Table 7:** Different approaches to define ontogenetic stages with different ecological roles in *Gonatus fabricii*. ML: mantle length, TL: trophic level, SCA: stomach content analysis, and SIA: stable isotope analysis.

Maturity Stages ^1^			‘Classical’ Ecology ^2^		This Study
Stage	Females	Males	Females	Males	
0 (juvenile)	ML 7–35 mm	ML 7–33 mm	Epipelagic paralarvae, ML 3.5–15 mm		Epipelagic squid with ML < 20 mm: prey mostly on crustaceans, especially copepods (SCA) or copepods and larger crustaceans in equal proportions (SIA); mass < 0.4 g
I (early immature)	ML 31–81 mm	ML 34–78 mm	Epipelagic, ML 15–60 mm		
II (late immature)	ML 72– 194 mm	ML 70– 121 mm	Mesopelagic ML 60–150 mm	Mesopelagic, ML 60–130 mm	Squid with ML 20–50 mm: mostly stay in the epipelagic layer, but occasionally exploit the whole water column; diet shifts from Copepoda to larger Crustacea, fish, and cephalopods; mass 0.7–5.8 g
III (early maturing)	ML 226– 306 mm	ML 113– 229 mm			
IV (late maturing)	ML 200– 288 mm	ML 198– 269 mm	Bathypelagic, ML > 150 mm	Bathypelagic, ML > 130 mm	Squid with ML > 50 mm: stay in the meso- and bathypelagic layers (occasionally might be recorded in the epipelagic layers, as they have daily vertical migrations); mostly prey on fish and cephalopods with a high proportion of cannibalism; mass > 6.3 g
V_1_ (pre-mature)	No data	ML 236 mm			
V_2_ (mature)	ML 221– 389 mm	ML 179– 320 mm			
V_3_ (sub-spent)	No data	ML 224– 325 mm	Bathypelagic gelatinous, ML > 200 mm ^3^		Bathypelagic gelatinous females with ML > 200 mm: non-feeding and almost non-moving; mass > 208.7 g ^4^
VI (spent)	ML 230– 322 mm	No data			

^1^ Nesis (1965), Kristensen (1981, 1984), Sennikov et al. (1989), Bjørke and Hansen (1996), Bjørke et al. (1997), Arkhipkin and Bjørke (1999), Zumholz and Frandsen (2006), Gardiner and Dick (2010b), Golikov et al. (2012, 2019b, 2021) and Golikov (2015) [36,37,39,40,41,42,43,46,126,127,128,129]; ^2^ Kristensen (1981, 1984), Bjørke and Hansen (1996), Arkhipkin and Bjørke (1999), Golikov et al. (2018) [27,36,39,40,42]; ^3^ non-gelatinous females exceeding ML 200 mm are common in the Baffin Bay and the Davis Strait (Kristensen 1984, Golikov et al. 2019b) [36,37]; ^4^ raw data on the mass of gelatinous females from Golikov et al. (2018, 2019b, 2021) [27,37,129].

## Data Availability

All relevant data are included in the paper and/or in the supplementary information file, or uploaded to the Zenodo data repository (https://doi.org/10.5281/zenodo.7113726).

## Supplementary Materials

The following supporting information can be downloaded at: https://www.mdpi.com/article/10.3390/ani12243548/s1, Table S1. All individuals of *Gonatus fabricii*, analysed for this study (*n* = 29): location, data, depth, sex, maturity stage, mantle length and upper beak measurements: rostrum length and crest length. ML: mantle length, URL: upper beak rostrum length, UCL: upper beak crest length, SIA: stable isotope analysis, n/a: no results, ---: not applicable. Table S2. Data on *Gonatus fabricii*, analysed by stable isotope analysis (*n* = 14), and each of the analysed upper beak subsections (*n* = 179): mantle length, sex, maturity stage, number and size of subsections, mass C:N ratio, values of *δ*^13^C and *δ*^15^N, estimated trophic level, and estimated mantle length and mass at given beak size. ML: mantle length, TL: trophic level, n/a: no results, ---: not applicable. Table S3. Equations to estimate mantle length and mass of *Gonatus fabricii* from upper beak rostrum length. Significant *p*-values are in bold. ML: mantle length, URL: upper beak rostrum length, *n*: sample size. Table S4. Values of *δ*^13^C and *δ*^15^N for the prey group sources used in Bayesian mixing model SIMMR 0.4.5. Values are mean ± SD. *N*: sample size, ---: not applicable. Table S5. Differences in values of *δ*^13^C and *δ*^15^N for the prey group sources used in Bayesian mixing model SIMMR 0.4.5. Kruskal–Wallis *H* and Dunn’s *Z* tests are provided in the table. Significant *p*-values are in bold. ---: not applicable. Table S6. Spearman’s rank correlation between *δ*^13^C and *δ*^15^N values in *Gonatus fabricii* at population and individual levels. Significant *p*-values are in bold. *n*: sample size. Table S7. Isotopic niche width, estimated in nicheROVER 1.1.0 for different upper beak subsections of *Gonatus fabricii*. Values are mean ± SD. *n*: sample size. Table S8. Isotopic niches metrics (TA, SEA_c_, and SEA_b_) and overlap in *Gonatus fabricii* across sexes, sizes, and studied areas, estimated in SIBER 2.1.6. SEA_b_ values are means ± SD. Significant *p*-values are in bold. Significant overlap values are in bold. ML: mantle length, *n*: sample size, ---: not applicable. Table S9. Differences in the predicted relative contribution of prey to the diet among different upper beak subsections of *Gonatus fabricii*, estimated in Bayesian mixing model SIMMR 0.4.5. *χ*^2^ test and Fisher’s exact test are provided in the table, above and below the diagonal, respectively. Significant *p*-values are in bold. ---: not applicable. Figure S1. Checking the data on *Gonatus fabricii* fitting to the prey group sources and trophic enrichment factors in SIMMR 0.4.5. Consumers (studied individuals; violet empty circles) and mean source signatures (see in-figure legend for reference) are shown. The exact values of sources and their standard deviations are in Table S3, and trophic enrichment factors and their standard deviations are in Materials and methods. Figure S2. Isotopic niche overlap among the upper beak subsections of *Gonatus fabricii*, estimated in nicheROVER 1.1.0.
