# Supplementary material for: Life History of the Arctic Squid Gonatus fabricii (Cephalopoda: Oegopsida) Reconstructed by Analysis of Individual Ontogenetic Stable Isotopic Trajectories
Source: Animals (Basel). 2022 Dec 15;12(24):3548. doi: 10.3390/ani12243548 (PMC9774963; doi:10.3390/ani12243548)

Table S1. All individuals of *Gonatus fabricii*, analysed for this study (*n* = 29): location, data, depth, sex, maturity stage, mantle length and upper beak measurements: rostrum length and crest length. ML – mantle length, URL – upper beak rostrum length, UCL – upper beak crest length, SIA – stable isotope analysis, n/a – no results, --- – not applicable.

Squid	Coordinates	Data	Depth, m	Sex	Maturity stage	ML, mm	Upper beak measurements		SIA
							URL, mm	UCL, mm	
F1	69.22° N<br>51.45° W	12/06/2017	407.0	Female	III	214	5.90	19.22	yes
F2	69.22° N<br>51.45° W	12/06/2017	407.0	Female	III	215	3.97[2]	18.87	yes
F3	67.46° N<br>62.05° W	08/10/2016	1110.0	Female	III	238	5.60[2]	20.49[2]	yes
F4	63.59° N<br>56.56° W	08/09/2016	1443.0	Female	III	249	5.49	22.02	yes
F5	69.47° N<br>65.07° W	15/10/2016	665.0	Female	III	257	5.48	19.23	yes
F6[1]	70.69° N<br>61.17° W	12/07/2019	1105.0	Female	VI	230	5.17	18.82[2]	yes
F7	69.06° N<br>51.62° W	08/06/2017	430.0	Female	II	205	5.15	18.18	no
F8	73.76° N<br>61.67° W	25/09/2017	584.0	Female	II	222	5.74	20.08	no
F9	69.28° N<br>52.79° W	09/08/2017	507.5	Female	III	212	4.60	18.53	no
F10	69.28° N<br>52.79° W	09/08/2017	507.5	Female	III	212	5.38	18.22	no
F11	67.05° N<br>58.56° W	18/10/2016	1237.0	Female	III	244	5.19[2]	21.70	no
F12	64.33° N<br>59.00° W	04/11/2016	522.0	Female	III	306	6.80	22.69	no
M1	69.22° N<br>51.45° W	12/06/2017	407.0	Male	III	216	5.26	18.26	yes
M2	64.02° N<br>57.27° W	17/10/2017	944.5	Male	III	221	4.53	17.55	yes
M3	72.01° N<br>71.04° W	13/10/2016	1326.0	Male	IV	269	6.57	20.86	yes
M4	61.99° N<br>50.01° W	25/07/2017	1232.0	Male	V_2	285	6.69	24.11	yes
M5	64.33° N<br>55.64° W	09/08/2019	1059.5	Male	V_2	252	5.48	19.85	yes
M6	67.92° N<br>58.62° W	29/08/2017	384.5	Male	V_3	284	5.37	22.01	yes
M7	64.39° N<br>55.10° W	29/10/2016	1049.0	Male	V_3	298	5.93	21.58[2]	yes
M8	67.24° N<br>60.38° W	07/10/2016	1148.0	Male	V_3	325	5.87	21.59	yes
M9	69.22° N<br>51.45° W	12/06/2017	407.0	Male	III	208	4.74	19.10	no
M10	69.28° N<br>52.79° W	09/08/2017	507.5	Male	III	218	4.87	18.81	no
M11	69.23° N<br>53.16° W	14/06/2016	489.5	Male	IV	241	6.09	19.81	no
M12	63.32° N<br>56.16° W	08/09/2016	1428.5	Male	IV	261	5.56	19.53	no
M13	69.23° N	14/06/2016	489.5	Male	V_1	236	5.96	20.97	no

	53.16° W								
M14	66.55° N 58.03° W	18/10/2016	957.0	Male	V_2	221	5.79[2]	21.11	no
M15	70.33° N 66.10° W	12/10/2016	1102.0	Male	V_2	241	5.86	19.49	no
M16	64.16° N 57.05° W	06/09/2016	769.0	Male	V_3	232	5.77	24.02	no
M17	67.46° N 62.05° W	08/10/2016	1110.0	Male	V_3	256	4.03	21.03	no

[1]gelatinous degeneration;

[2]damaged, not used for equation.

Table S2. Data on *Gonatus fabricii*, analysed by stable isotope analysis (*n* = 14), and each of the analysed upper beak subsections (*n* = 179): mantle length, sex, maturity stage, number and size of subsections, mass C:N ratio, values of δ^{13}C and δ^{15}N, estimated trophic level, and estimated mantle length and mass at given beak size. ML – mantle length, TL – estimated trophic level, n/a – no results, --- – not applicable.

Squid	Subsections																	
F1	Number	1	2	3	4	5	6	7	8	9	10	11	12	13	14	15	16	17
	Size, mm	0.91	0.91	0.91	0.91	0.91	0.91	0.91	0.91	0.91	1.64	1.64	1.64	1.64	---	---	---	---
	C:N ratio	3.61	3.64	3.48	3.40	3.20	3.47	3.14	3.16	3.21	3.14	3.20	3.39	3.78	---	---	---	---
	δ^{13}C, ‰	–20.47	–20.28	–19.95	–19.78	–18.89	–18.74	–18.60	–18.76	–18.80	–18.76	–18.79	–18.73	–18.65	---	---	---	---
	δ^{15}N, ‰	9.01	7.94	9.89	10.07	12.26	11.70	13.33	13.36	13.32	13.54	13.49	12.94	13.08	---	---	---	---
	TL	3.29	3.01	3.52	3.57	4.15	4.00	4.43	4.44	4.43	4.48	4.47	4.33	4.36	---	---	---	---
	Est. ML, mm	7.0	11.7	17.5	24.0	31.3	39.2	47.6	56.6	66.0	84.0	103.2	123.6	145.0	167.4	---	---	---
	Est. mass, g	0.03	0.11	0.32	0.72	1.42	2.53	4.18	6.51	9.67	18.00	30.61	48.68	73.48	106.37	---	---	---
F2	Number	1	2	3	4	5	6	7	8	9	10	11	12	13	14	15	16	17
	Size, mm	0.90	0.90	0.90	0.90	0.90	0.90	0.90	0.90	0.90	1.63	1.63	1.63	1.63	---	---	---	---
	C:N ratio	n/a	n/a	n/a	n/a	n/a	n/a	n/a	n/a	n/a	n/a	n/a	n/a	3.69	---	---	---	---
	δ^{13}C, ‰	–20.41	–20.25	–20.47	–20.13	–20.12	–20.01	–20.00	–19.44	–19.56	–19.74	–19.72	–19.72	–19.70	---	---	---	---
	δ^{15}N, ‰	8.63	10.17	8.79	9.32	11.68	10.44	11.76	13.53	12.85	13.21	13.16	13.04	12.21	---	---	---	---
	TL	3.19	3.60	3.23	3.37	4.00	3.67	4.01	4.48	4.30	4.40	4.39	4.35	4.13	---	---	---	---
	Est. ML, mm	7.0	11.6	17.3	23.7	30.9	38.7	47.0	55.8	65.0	82.8	101.9	122.1	143.3	165.5	---	---	---
	Est. mass, g	0.03	0.11	0.31	0.70	1.37	2.45	4.04	6.28	9.32	17.38	29.61	47.15	71.24	103.21	---	---	---
F3	Number	1	2	3	4	5	6	7	8	9	10	11	12	13	14	15	16	17
	Size, mm	0.95	0.95	0.95	0.95	0.95	0.95	0.95	0.95	0.95	0.95	1.91	1.91	1.91	1.91	1.91	---	---
	C:N ratio	3.54	3.43	3.41	3.31	n/a	3.47	3.75	n/a	n/a	3.19	3.17	n/a	3.13	3.12	3.52	---	---
	δ^{13}C, ‰	–20.52	–20.37	–19.76	–19.47	n/a	–20.20	–19.35	n/a	n/a	–19.23	–19.29	n/a	–19.06	–18.92	–19.10	---	---
	δ^{15}N, ‰	10.74	11.71	12.84	12.76	14.50	10.32	14.54	n/a	n/a	15.43	15.81	n/a	16.74	16.39	15.04	---	---
	TL	3.75	4.00	4.30	4.28	4.74	3.64	4.75	n/a	n/a	4.98	5.08	n/a	5.33	5.23	4.88	---	---
	Est. ML, mm	7.2	12.2	18.3	25.3	33.0	41.4	50.3	59.8	69.8	80.2	102.5	126.3	151.5	178.0	205.7	---	---
	Est. mass, g	0.03	0.12	0.36	0.82	1.63	2.91	4.82	7.52	11.19	16.01	30.06	51.46	82.20	124.50	180.71	---	---
F4	Number	1	2	3	4	5	6	7	8	9	10	11	12	13	14	15	16	17
	Size, mm	1.0	1.0	1.0	1.0	1.0	1.0	1.0	1.0	1.0	1.0	2.0	2.0	2.0	2.0	---	---	---
	C:N ratio	n/a	n/a	3.72	3.24	3.14	n/a	n/a	3.16	3.16	n/a	n/a	n/a	n/a	3.21	---	---	---
	δ^{13}C, ‰	n/a	n/a	–19.33	–19.37	–18.62	n/a	n/a	–18.94	–18.96	n/a	n/a	n/a	n/a	–19.27	---	---	---
	δ^{15}N, ‰	n/a	6.41	13.91	15.49	11.45	n/a	n/a	11.76	11.82	n/a	11.07	11.55	10.75	15.26	---	---	---
	TL	n/a	3.03	5.00	5.42	4.35	n/a	n/a	4.44	4.45	n/a	4.25	4.38	4.17	5.36	---	---	---
	Est. ML, mm	7.4	12.8	19.3	26.8	35.2	44.2	53.8	64.0	74.7	85.9	109.7	135.1	162.0	190.3	219.9	---	---
	Est. mass, g	0.04	0.14	0.41	0.96	1.91	3.44	5.72	8.94	13.32	19.09	35.80	61.22	97.74	147.94	214.62	---	---
F5	Number	1	2	3	4	5	6	7	8	9	10	11	12	13	14	15	16	17

	Size, mm	0.97	0.97	0.97	0.97	0.97	0.97	0.97	0.97	0.97	1.74	1.74	1.74	1.74	---	---	---	---
	C:N ratio	n/a	n/a	n/a	3.34	3.38	3.19	3.14	3.16	3.16	n/a	3.19	3.30	3.44	---	---	---	---
	δ^{13}C, ‰	n/a	n/a	n/a	–19.77	–19.68	–19.00	–19.03	–19.23	–19.28	n/a	–19.10	–19.08	–19.16	---	---	---	---
	δ^{15}N, ‰	n/a	n/a	n/a	13.68	15.10	15.42	16.22	16.11	16.09	15.75	16.30	16.12	15.28	---	---	---	---
	TL	n/a	n/a	n/a	4.52	4.89	4.98	5.19	5.16	5.15	5.07	5.21	5.16	4.94	---	---	---	---
	Est. ML, mm	7.3	12.4	18.7	25.9	33.9	42.5	51.7	61.5	71.8	91.3	112.3	134.4	157.7	182.1	---	---	---
	Est. mass, g	0.03	0.13	0.38	0.87	1.74	3.11	5.17	8.07	12.01	22.35	38.02	60.46	91.25	132.08	---	---	---
F6[1]	Number	1	2	3	4	5	6	7	8	9	10	11	12	13	14	15	16	17
	Size, mm	0.94	0.94	0.94	0.94	0.94	0.94	0.94	0.94	0.94	0.94	1.88	1.88	1.88	1.88	1.88	---	---
	C:N ratio	3.60	3.39	3.4	3.16	3.13	3.11	3.11	3.07	3.05	3.15	3.16	3.16	3.15	3.16	3.22	---	---
	δ^{13}C, ‰	–20.24	–19.79	–18.89	–19.26	–19.21	–19.16	–19.12	–19.19	–19.15	–19.13	–19.22	–19.11	–18.85	–18.61	–18.53	---	---
	δ^{15}N, ‰	11.17	11.86	13.08	13.83	14.39	15.66	15.52	16.09	16.12	15.67	15.59	16.54	16.54	16.13	16.75	---	---
	TL	3.65	3.83	4.15	4.35	4.50	4.83	4.80	4.94	4.95	4.83	4.81	5.06	5.06	4.96	5.12	---	---
	Est. ML, mm	7.2	12.1	18.1	25.0	32.6	40.8	49.7	59.0	68.8	79.1	100.9	124.2	148.9	174.8	202.0	---	---
	Est. mass, g	0.03	0.12	0.35	0.79	1.57	2.81	4.66	7.26	10.79	15.44	28.90	49.33	78.66	118.96	172.47	---	---
M1	Number	1	2	3	4	5	6	7	8	9	10	11	12	13	14	15	16	17
	Size, mm	0.99	0.99	0.99	0.99	0.99	0.99	0.99	0.99	1.99	1.99	1.99	1.99	---	---	---	---	---
	C:N ratio	3.56	n/a	3.50	3.25	3.22	n/a	3.23	n/a	3.27	3.25	3.57	3.50	---	---	---	---	---
	δ^{13}C, ‰	–20.13	n/a	–19.55	–18.53	–18.63	n/a	–18.95	n/a	–18.86	–18.75	–18.82	–19.08	---	---	---	---	---
	δ^{15}N, ‰	8.94	n/a	9.77	11.65	13.15	n/a	13.51	n/a	13.83	14.35	13.82	13.45	---	---	---	---	---
	TL	3.27	n/a	3.49	3.99	4.38	n/a	4.48	n/a	4.56	4.70	4.56	4.46	---	---	---	---	---
	Est. ML, mm	7.4	12.7	19.1	26.5	34.7	43.6	53.1	63.1	84.8	108.2	133.3	159.8	---	---	---	---	---
	Est. mass, g	0.04	0.14	0.41	0.97	1.94	3.49	5.82	9.11	19.53	36.72	62.96	100.73	---	---	---	---	---
M2	Number	1	2	3	4	5	6	7	8	9	10	11	12	13	14	15	16	17
	Size, mm	0.97	0.97	0.97	0.97	0.97	0.97	0.97	0.97	1.93	1.93	1.93	---	---	---	---	---	---
	C:N ratio	3.61	3.52	3.38	3.35	3.21	3.09	3.16	3.22	3.14	3.27	3.49	---	---	---	---	---	---
	δ^{13}C, ‰	–21.18	–20.85	–20.47	–19.99	–19.28	–19.37	–19.40	–19.47	–19.45	–19.45	–19.59	---	---	---	---	---	---
	δ^{15}N, ‰	6.12	6.30	7.03	8.22	11.02	11.68	11.72	10.69	12.24	12.36	11.98	---	---	---	---	---	---
	TL	2.95	3.00	3.19	3.51	4.24	4.42	4.43	4.15	4.56	4.59	4.49	---	---	---	---	---	---
	Est. ML, mm	7.3	12.4	18.7	25.9	33.9	42.5	51.7	61.5	82.4	105.0	129.2	154.9	---	---	---	---	---
	Est. mass, g	0.03	0.14	0.39	0.91	1.82	3.27	5.44	8.51	18.14	34.00	58.16	92.88	---	---	---	---	---
M3	Number	1	2	3	4	5	6	7	8	9	10	11	12	13	14	15	16	17
	Size, mm	0.92	0.92	0.92	0.92	0.92	0.92	0.92	0.92	0.92	0.92	1.84	1.84	1.84	1.84	---	---	---
	C:N ratio	3.66	3.64	3.58	3.40	3.30	3.12	3.24	3.11	3.13	3.18	3.18	3.33	3.49	3.86	---	---	---
	δ^{13}C, ‰	–20.98	–20.88	–20.96	–20.19	–20.14	–19.58	–19.64	–19.41	–19.63	–19.64	–19.56	–19.66	–19.98	–19.78	---	---	---
	δ^{15}N, ‰	9.53	9.49	10.58	10.62	12.58	13.69	14.03	14.72	14.48	15.43	14.92	14.80	14.56	13.82	---	---	---
	TL	3.77	3.76	4.05	4.06	4.57	4.87	4.95	5.14	5.07	5.32	5.19	5.16	5.09	4.90	---	---	---
	Est. ML, mm	7.1	11.8	17.7	24.3	31.7	39.8	48.3	57.4	66.9	76.9	98.0	120.7	144.6	169.8	196.1	---	---
	Est. mass, g	0.03	0.12	0.34	0.77	1.54	2.75	4.56	7.12	10.60	15.18	28.46	48.69	77.79	117.85	171.13	---	---
M4	Number	1	2	3	4	5	6	7	8	9	10	11	12	13	14	15	16	17

	Size, mm	0.96	0.96	0.96	0.96	0.96	0.96	0.96	0.96	0.96	0.96	0.96	1.75	1.75	1.75	1.75	1.75	---
	C:N ratio	3.57	3.52	3.46	n/a	3.35	3.18	3.13	3.18	3.22	3.21	3.16	3.21	3.25	3.45	3.44	3.71	---
	δ^{13}C, ‰	–20.35	–20.04	–19.72	n/a	–19.13	–18.50	–18.80	–19.05	–18.94	–18.83	–18.46	–18.17	–18.32	–18.98	–19.09	–19.33	---
	δ^{15}N, ‰	5.56	5.74	6.51	n/a	8.19	9.98	10.74	10.70	10.70	11.17	11.54	11.22	11.11	10.78	10.50	10.07	---
	TL	3.02	3.06	3.27	n/a	3.71	4.18	4.38	4.37	4.37	4.49	4.59	4.50	4.48	4.39	4.32	4.20	---
	Est. ML, mm	7.3	12.3	18.5	25.6	33.4	41.9	51.0	60.7	70.8	81.4	92.4	113.5	135.9	159.4	184.0	209.5	236.1
	Est. mass, g	0.03	0.13	0.38	0.88	1.76	3.16	5.25	8.21	12.25	17.57	24.40	41.57	66.21	100.06	145.01	203.10	276.48
M5	Number	1	2	3	4	5	6	7	8	9	10	11	12	13	14	15	16	17
	Size, mm	0.97	0.97	0.97	0.97	0.97	0.97	0.97	0.97	0.97	1.75	1.75	1.75	1.75	1.75	---	---	---
	C:N ratio	n/a	n/a	n/a	n/a	n/a	n/a	n/a	n/a	n/a	n/a	n/a	n/a	n/a	n/a	---	---	---
	δ^{13}C, ‰	–19.35	–19.26	–19.63	–18.98	–19.11	–18.91	–19.11	–19.13	–19.18	–19.36	–19.43	–19.28	–19.09	–19.24	---	---	---
	δ^{15}N, ‰	8.39	8.28	6.38	8.49	10.19	9.88	11.80	11.72	11.78	12.33	12.28	11.49	11.07	10.14	---	---	---
	TL	3.55	3.52	3.02	3.58	4.02	3.94	4.45	4.43	4.44	4.59	4.57	4.37	4.25	4.01	---	---	---
	Est. ML, mm	7.3	12.4	18.7	25.9	33.9	42.5	51.7	61.5	71.8	91.4	112.5	134.8	158.3	182.8	---	---	---
	Est. mass, g	0.03	0.14	0.39	0.91	1.82	3.27	5.44	8.51	12.69	23.77	40.64	64.89	98.28	142.69	---	---	---
M6	Number	1	2	3	4	5	6	7	8	9	10	11	12	13	14	15	16	17
	Size, mm	0.99	0.99	0.99	0.99	0.99	0.99	0.99	0.99	0.99	0.99	1.98	1.98	1.98	1.98	1.98	---	---
	C:N ratio	3.54	3.63	3.62	3.40	3.28	3.38	3.19	3.14	3.26	3.22	3.21	3.13	3.20	3.23	3.65	---	---
	δ^{13}C, ‰	–20.75	–20.40	–20.35	–19.70	–19.39	–19.73	–19.28	–19.42	–19.43	–19.54	–19.55	–19.45	–19.31	–19.20	–19.64	---	---
	δ^{15}N, ‰	11.09	11.74	12.49	13.39	14.07	14.30	14.97	15.16	15.45	15.57	15.53	16.15	16.58	16.42	14.27	---	---
	TL	3.84	4.01	4.21	4.44	4.62	4.68	4.86	4.91	4.99	5.02	5.01	5.17	5.28	5.24	4.68	---	---
	Est. ML, mm	7.4	12.7	19.1	26.5	34.7	43.6	53.1	63.1	73.7	84.8	108.2	133.3	159.8	187.7	216.9	---	---
	Est. mass, g	0.04	0.14	0.41	0.97	1.94	3.49	5.82	9.11	13.60	19.53	36.72	62.96	100.73	152.76	222.01	---	---
M7	Number	1	2	3	4	5	6	7	8	9	10	11	12	13	14	15	16	17
	Size, mm	0.98	0.98	0.98	0.98	0.98	0.98	0.98	0.98	0.98	0.98	0.98	1.80	1.80	1.80	1.80	1.80	1.80
	C:N ratio	n/a	n/a	n/a	3.27	n/a	n/a	3.13	n/a	n/a	n/a	n/a	n/a	n/a	n/a	3.29	n/a	3.23
	δ^{13}C, ‰	n/a	n/a	n/a	–19.29	n/a	n/a	–19.10	n/a	n/a	n/a	n/a	n/a	n/a	n/a	–19.13	n/a	–19.46
	δ^{15}N, ‰	n/a	10.98	10.30	11.91	12.29	12.73	15.31	14.80	15.39	15.17	14.65	n/a	14.55	14.84	15.02	n/a	15.25
	TL	n/a	4.23	4.05	4.48	4.58	4.69	5.37	5.24	5.39	5.34	5.20	n/a	5.17	5.25	5.29	n/a	5.36
	Est. ML, mm	7.4	12.5	18.9	26.2	34.3	43.0	52.4	62.3	72.7	83.6	95.0	116.9	140.1	164.5	190.0	216.6	244.1
	Est. mass, g	0.04	0.14	0.40	0.94	1.88	3.38	5.62	8.81	13.14	18.86	26.20	44.84	71.65	108.57	157.68	221.22	301.57
M8	Number	1	2	3	4	5	6	7	8	9	10	11	12	13	14	15	16	17
	Size, mm	0.96	0.96	0.96	0.96	0.96	0.96	0.96	0.96	0.96	0.96	0.96	1.75	1.75	1.75	1.75	1.75	1.75
	C:N ratio	3.59	n/a	n/a	3.34	3.22	3.17	3.09	3.15	3.16	n/a	n/a	n/a	3.19	3.16	3.14	3.45	3.72
	δ^{13}C, ‰	–20.75	n/a	n/a	–19.94	–19.46	–19.32	–19.56	–19.75	–19.71	n/a	n/a	n/a	–19.44	–19.55	–19.65	–19.78	–19.94
	δ^{15}N, ‰	10.84	10.69	11.41	12.20	13.37	13.99	15.10	14.65	14.79	n/a	n/a	n/a	14.92	14.72	14.80	14.18	13.50
	TL	3.77	3.73	3.92	4.13	4.44	4.60	4.89	4.78	4.81	n/a	n/a	n/a	4.85	4.80	4.82	4.65	4.47
	Est. ML, mm	7.3	12.3	18.5	25.6	33.4	41.9	51.0	60.7	70.8	81.4	92.4	113.5	135.9	159.4	184.0	209.5	236.1
	Est. mass, g	0.03	0.13	0.38	0.88	1.76	3.16	5.25	8.21	12.25	17.57	24.40	41.57	66.21	100.06	145.01	203.10	276.48

[1]gelatinous degeneration.

Table S3. Equations to estimate mantle length and mass of *Gonatus fabricii* from upper beak rostrum length. Significant *p*-values are in **bold**. ML – mantle length, URL – upper beak rostrum length, *n* – sample size.

Area	ML	Mass	Source
Arctic (in general)	ML = 30.91*URL$^{1.06}$, n = 208, R^2 = 0.88, $\mathbf{p = 0.0001}$	Mass = 1.50*URL$^{2.66}$, n = 208, R^2 = 0.89, $\mathbf{p = 0.0001}$	This study
West Greenland	ML = 26.86*URL$^{1.18}$, n = 124, R^2 = 0.93, $\mathbf{p = 0.0001}$	Mass = 0.93*URL$^{3.00}$, n = 124, R^2 = 0.95, $\mathbf{p = 0.0001}$	This study
East Greenland	ML = 27.79*URL$^{0.96}$, n = 29, R^2 = 0.93, $\mathbf{p = 0.0100}$	Mass = 1.73*URL$^{2.24}$, n = 29, R^2 = 0.97, $\mathbf{p = 0.0090}$	Golikov et al. 2018
Barents Sea	ML = 38.22*URL$^{0.88}$, n = 67, R^2 = 0.95, $\mathbf{p = 0.0100}$	Mass = 2.71*URL$^{2.16}$, n = 67, R^2 = 0.97, $\mathbf{p = 0.0090}$	Golikov et al. 2018

Table S4. Values of $\delta^{13}C$ and $\delta^{15}N$ for the prey group sources used in Bayesian mixing model SIMMR 0.4.5. Values are mean ± SD. *n* – sample size, **---** – not applicable.

Prey sources[1]		Subsections 1–3	Subsections 4–5	Subsections 7–9; subsection 10
Copepoda	$\delta^{13}C$, ‰	*n* = 75, –20.93 ± 1.00	*n* = 75, –20.93 ± 1.00	*n* = 75, –20.93 ± 1.00
	$\delta^{15}N$, ‰	*n* = 84, 8.49 ± 1.19	*n* = 84, 8.49 ± 1.19	*n* = 84, 8.49 ± 1.19
Euphausiacea	$\delta^{13}C$, ‰	*n* = 34, –20.83 ± 1.13	*n* = 34, –20.83 ± 1.13	*n* = 34, –20.83 ± 1.13
	$\delta^{15}N$, ‰	*n* = 34, 9.71 ± 1.21	*n* = 34, 9.71 ± 1.21	*n* = 34, 9.71 ± 1.21
Shrimps	$\delta^{13}C$, ‰	*n* = 22, –17.90 ± 0.98	*n* = 22, –17.90 ± 0.98	*n* = 22, –17.90 ± 0.98
	$\delta^{15}N$, ‰	*n* = 22, 11.48 ± 1.65	*n* = 22, 1148 ± 1.65	*n* = 22, 1148 ± 1.65
Chaetognatha	$\delta^{13}C$, ‰	*n* = 35, –18.56 ± 7.64	*n* = 35, –18.56 ± 7.64	*n* = 35, –18.56 ± 7.64
	$\delta^{15}N$, ‰	*n* = 36, 11.91 ± 1.93	*n* = 36, 11.91 ± 1.93	*n* = 36, 11.91 ± 1.93
Cephalopoda	$\delta^{13}C$, ‰	---[2]	*n* = 35, –20.28 ± 0.69	*n* = 57, –19.98 ± 0.69
	$\delta^{15}N$, ‰	---[2]	*n* = 35, 12.84 ± 1.57	*n* = 57, 14.48 ± 2.66
Fishes	$\delta^{13}C$, ‰	---[2]	*n* = 30, –20.21 ± 0.66	*n* = 51, –20.11 ± 0.80
	$\delta^{15}N$, ‰	---[2]	*n* = 30, 11.61 ± 1.12	*n* = 51, 11.87 ± 1.33
Cephalopoda + fishes	$\delta^{13}C$, ‰	*n* = 39, –20.44 ± 0.67	---[2]	---[2]
	$\delta^{15}N$, ‰	*n* = 39, 11.73 ± 1.22	---[2]	---[2]

[1]Pomerleau et al. (2011, 2016) [97,110], Hansen et al. (2012) [96], Agersted et al. (2014) [111], Linnebjerg et al. (2016) [94], Golikov et al. (2018) [27], Grigor et al. (2020) [101] and this study; [2]source values are not significantly different, thus, Cephalopoda and fishes are combined in this case.

Table S5. Differences in values of δ^{13}C and δ^{15}N for the prey group sources used in Bayesian mixing model SIMMR 0.4.5. Kruskal–Wallis *H* and Dunn's *Z* tests are provided in the table. Significant *p*-values are in **bold**. **---** – not applicable.

δ^{13}C						
Subsections 1–3: $H_{4,205}$ = 84.91, ***p* < 0.0001**						
Prey source	Copepoda	Euphausiacea	Shrimps	Chaetognatha	Cephalopoda + Fishes[1]	
Copepoda	---	*Z* = 0.37	*Z* = 8.10.5	*Z* = 5.40	*Z* = 2.18	
Euphausiacea	*p* = 0.71	---	*Z* = 6.90	*Z* = 4.27	*Z* = 1.50	
Shrimps	***p* < 0.0001**	***p* < 0.0001**	---	*Z* = 3.16	*Z* = 5.75	
Chaetognatha	***p* < 0.0001**	***p* < 0.0001**	***p* = 0.0016**	---	*Z* = 2.90	
Cephalopoda + Fishes[1]	***p* = 0.0294**	*p* = 0.13	***p* < 0.0001**	***p* = 0.0038**	---	
Subsections 4–5: $H_{5,231}$ = 92.79, ***p* < 0.0001**						
Prey source	Copepoda	Euphausiacea	Shrimps	Chaetognatha	Cephalopoda	Fishes
Copepoda	---	*Z* = 0.45	*Z* = 8.46	*Z* = 5.68	*Z* = 2.95	*Z* = 3.00
Euphausiacea	*p* = 0.65	---	*Z* = 7.16	*Z* = 4.45	*Z* = 2.13	*Z* = 2.19
Shrimps	***p* < 0.0001**	***p* < 0.0001**	---	*Z* = 3.27	*Z* = 5.32	*Z* = 5.02
Chaetognatha	***p* < 0.0001**	***p* < 0.0001**	***p* = 0.0011**	---	*Z* = 2.34	*Z* = 2.10
Cephalopoda	***p* = 0.0032**	***p* = 0.0339**	***p* < 0.0001**	***p* = 0.0195**	---	*Z* = 0.15
Fishes	***p* = 0.0030**	***p* = 0.0284**	***p* < 0.0001**	***p* = 0.0361**	*p* = 0.88	---
Subsections 7–9 and subsection 10: $H_{5,274}$ = 106.30, ***p* < 0.0001**						
Prey source	Copepoda	Euphausiacea	Shrimps	Chaetognatha	Cephalopoda	Fishes
Copepoda	---	*Z* = 0.50	*Z* = 8.93	*Z* = 5.79	*Z* = 5.14	*Z* = 3.90
Euphausiacea	*p* = 0.62	---	*Z* = 754	*Z* = 4.49	*Z* = 3.69	*Z* = 2.73
Shrimps	***p* < 0.0001**	***p* < 0.0001**	---	*Z* = 3.61	*Z* = 5.03	*Z* = 5.72
Chaetognatha	***p* < 0.0001**	***p* < 0.0001**	***p* = 0.0003**	---	*Z* = 1.31	*Z* = 2.17
Cephalopoda	***p* < 0.0001**	***p* = 0.0002**	***p* < 0.0001**	*p* = 0.19	---	*Z* = 1.01
Fishes	***p* < 0.0001**	***p* = 0.0063**	***p* < 0.0001**	***p* = 0.0298**	*p* = 0.31	---

δ^{15}N						
Subsections 1–3: $H_{4,215}$ = 119.70, ***p* < 0.0001**						
Prey source	Copepoda	Euphausiacea	Shrimps	Chaetognatha	Cephalopoda + Fishes[1]	
Copepoda	---	*Z* = 3.18	*Z* = 6.24	*Z* = 8.30	*Z* = 8.53	
Euphausiacea	***p* = 0.0017**	---	*Z* = 3.13	*Z* = 4.25	*Z* = 4.33	
Shrimps	***p* < 0.0001**	***p* = 0.0017**	---	*Z* = 0.59	*Z* = 0.60	
Chaetognatha	***p* < 0.0001**	***p* < 0.0001**	*p* = 0.56	---	*Z* = 0.001	
Cephalopoda + Fishes[1]	***p* < 0.0001**	***p* < 0.0001**	*p* = 0.55	*p* = 0.99	---	
Subsections 4–5: $H_{5,241}$ = 145.50, ***p* < 0.0001**						
Prey source	Copepoda	Euphausiacea	Shrimps	Chaetognatha	Cephalopoda	Fishes
Copepoda	---	*Z* = 2.84	*Z* = 5.92	*Z* = 8.04	*Z* = 9.72	*Z* = 7.16
Euphausiacea	***p* = 0.0045**	---	*Z* = 3.07	*Z* = 4.28	*Z* = 5.72	*Z* = 3.77
Shrimps	***p* < 0.0001**	***p* = 0.0022**	---	*Z* = 0.68	*Z* = 1.97	*Z* = 0.38
Chaetognatha	***p* < 0.0001**	***p* < 0.0001**	*p* = 0.50	---	*Z* = 1.49	*Z* = 0.32
Cephalopoda	***p* < 0.0001**	***p* < 0.0001**	***p* = 0.0484**	*p* = 0.14	---	*Z* = 2.01
Fishes	***p* < 0.0001**	***p* = 0.0002**	*p* = 0.71	*p* = 0.75	***p* = 0.0428**	---
Subsections 7–9 and subsection 10: $H_{5,284}$ = 184.30, ***p* < 0.0001**						
Prey source	Copepoda	Euphausiacea	Shrimps	Chaetognatha	Cephalopoda	Fishes
Copepoda	---	*Z* = 2.51	*Z* = 5.38	*Z* = 7.38	*Z* = 12.35	*Z* = 8.28
Euphausiacea	***p* < 0.0121**	---	*Z* = 2.85	*Z* = 4.01	*Z* = 7.43	*Z* = 4.34
Shrimps	***p* < 0.0001**	***p* = 0.0044**	---	*Z* = 0.67	*Z* = 3.31	*Z* = 0.71
Chaetognatha	***p* < 0.0001**	***p* < 0.0001**	*p* = 0.51	---	*Z* = 3.06	*Z* = 0.003
Cephalopoda	***p* < 0.0001**	***p* < 0.0001**	***p* = 0.0009**	***p* = 0.0023**	---	*Z* = 3.37
Fishes	***p* < 0.0001**	***p* < 0.0001**	*p* = 0.48	*p* = 0.99	***p* = 0.0008**	---

[1]source values are not significantly different, thus, Cephalopoda and fishes are combined in this case.

Table S6. Spearman's rank correlation between $\delta^{13}C$ and $\delta^{15}N$ values in *Gonatus fabricii* at population and individual levels. Significant *p*-values are in **bold**. *n* – sample size.

Parameter	Population level		Individual level						
	West Greenland, East Greenland and the Barents Sea	West Greenland	F1	F2	F6	M2	M3	M5	M6
n[1]	118	57	13	13	15	11	14	14	15
Spearman *r*	0.44	0.71	0.65	0.88	0.75	0.68	0.77	-0.06	0.73
p	**< 0.0001**	**< 0.0001**	**0.0165**	**< 0.0001**	**0.0014**	**0.0208**	**0.0012**	0.83	**0.0021**

[1]individuals at population level, and upper beak subsections at individual level.

Table S7. Isotopic niche width, estimated in nicheROVER 1.1.0 for different upper beak subsections of *Gonatus fabricii*. Values are mean ± SD. *n* – sample size.

Parameter	Subsection													
	1	2	3	4	5	6	7	8	9	10	11	12	13	14
n	11	12	13	13	14	12	13	12	13	12	13	10	12	9
Niche width	2.68 ± 0.78	3.53 ± 1.21	6.18 ± 1.95	4.51 ± 1.28	2.65 ± 0.79	4.28 ± 1.38	2.39 ± 0.63	1.54 ± 0.50	1.45 ± 0.45	1.74 ± 0.60	2.08 ± 0.67	3.04 ± 1.06	3.50 ± 1.21	2.82 ± 1.06

Table S8. Isotopic niches metrics (TA, SEA_c and SEA_b) and overlap in *Gonatus fabricii* across sexes, sizes and studied areas, estimated in SIBER 2.1.6. SEA_b values are means ± SD. Significant *p*-values are in **bold**. Significant overlap values are in **bold**. ML – mantle length, *n* – sample size, **---** – not applicable.

Parameter	Arctic[1] (ML 20–50 mm)		Arctic[1] (ML > 50 mm)		West Greenland (ML > 50 mm)		Females, Arctic[1]		Males, Arctic[1]	
	Females	Males	Females	Males	Females	Males	ML 20–50 mm	ML > 50 mm	ML 20–50 mm	ML > 50 mm
n	18	11	43	42	20	20	18	43	11	42
TA	1.97	2.10	5.08	5.62	2.17	1.56	1.97	4.33	2.10	5.62
SEA_c	0.99	1.14	1.62	1.51	0.87	0.63	1.09	1..57	1.14	1.51
SEA_b	1.00 ± 0.25	1.20 ± 0.40	1.63 ± 0.26	1.51 ± 0.24	0.88 ± 0.21	0.63 ± 0.15	1.11 ± 0.29	1.56 ± 0.28	1.20 ± 0.42	1.51 ± 0.23
Group 1 *p*-values	---	0.66	---	0.36	---	0.15	---	0.88	---	0.79
Group 2 *p*-values	0.34	---	0.64	---	0.85	---	0.12	---	0.21	---
Overlap, %	**80.47**	**73.08**	**80.10**	**86.39**	**71.61**	**99.16**	0.31	0.21	19.98	14.00

[1]West Greenland, East Greenland and the Barents Sea.

Table S9. Differences in predicted relative contribution of prey to the diet among different upper beak subsections of *Gonatus fabricii*, estimated in Bayesian mixing model SIMMR 0.4.5. χ^2 test and Fisher's exact test are provided in the table, above and below the diagonal, respectively. Significant *p*-values are in **bold**. **---** – not applicable.

Crustacea vs. Chaetognatha vs. Cephalopoda + fishes: $\chi^2 = 6.56$, *d .f.* = 6, $p = 0.36$					Copepoda vs. Euphausiacea vs. shrimps: $\chi^2 = 34.64$, *d .f.* = 4, $p <$ **0.0001**				
Subsections	1–3	4–5	7–9	10	Subsections	1–3	4–5	7–9	10
1–3	---	$\chi^2 = 0.29$, *d .f.* = 2, $p = 0.87$	$\chi^2 = 1.17$, *d .f.* = 2, $p = 0.56$	$\chi^2 = 2.90$, *d .f.* = 2, $p = 0.23$	1–3	---	$\chi^2 = 19.73$, *d .f.* = 2, $p <$ **0.0001**	$\chi^2 = 28.23$, *d .f.* = 2, $p <$ **0.0001**	$\chi^2 = 10.48$, *d .f.* = 2, $p =$ **0.0053**
4–5	Fisher's $p = 0.87$	---	$\chi^2 = 2.26$, *d .f.* = 2, $p = 0.32$	$\chi^2 = 2.59$, *d .f.* = 2, $p = 0.27$	4–5	Fisher's $p <$ **0.0001**	---	$\chi^2 = 1.78$, *d .f.* = 2, $p = 0.41$	$\chi^2 = 1.19$, *d .f.* = 2, $p = 0.55$
7–9	Fisher's $p = 0.53$	Fisher's $p = 0.33$	---	$\chi^2 = 2.52$, *d .f.* = 2, $p = 0.28$	7–9	Fisher's $p <$ **0.0001**	Fisher's $p = 0.45$	---	$\chi^2 = 5.20$, *d .f.* = 2, $p = 0.07$
10	Fisher's $p = 0.23$	Fisher's $p = 0.29$	Fisher's $p = 0.32$	---	10	Fisher's $p =$ **0.0062**	Fisher's $p = 0.59$	Fisher's $p = 0.09$	---

Figure S1. Checking the data on *Gonatus fabricii* fitting to the prey group sources and trophic enrichment factors in SIMMR 0.4.5. Consumers (studied individuals; violet empty circles) and mean source signatures (see in-figure legend for reference) are shown. Exact values of sources and their standard deviations are in Table S3, trophic enrichment factors and their standard deviations are in Materials and methods.

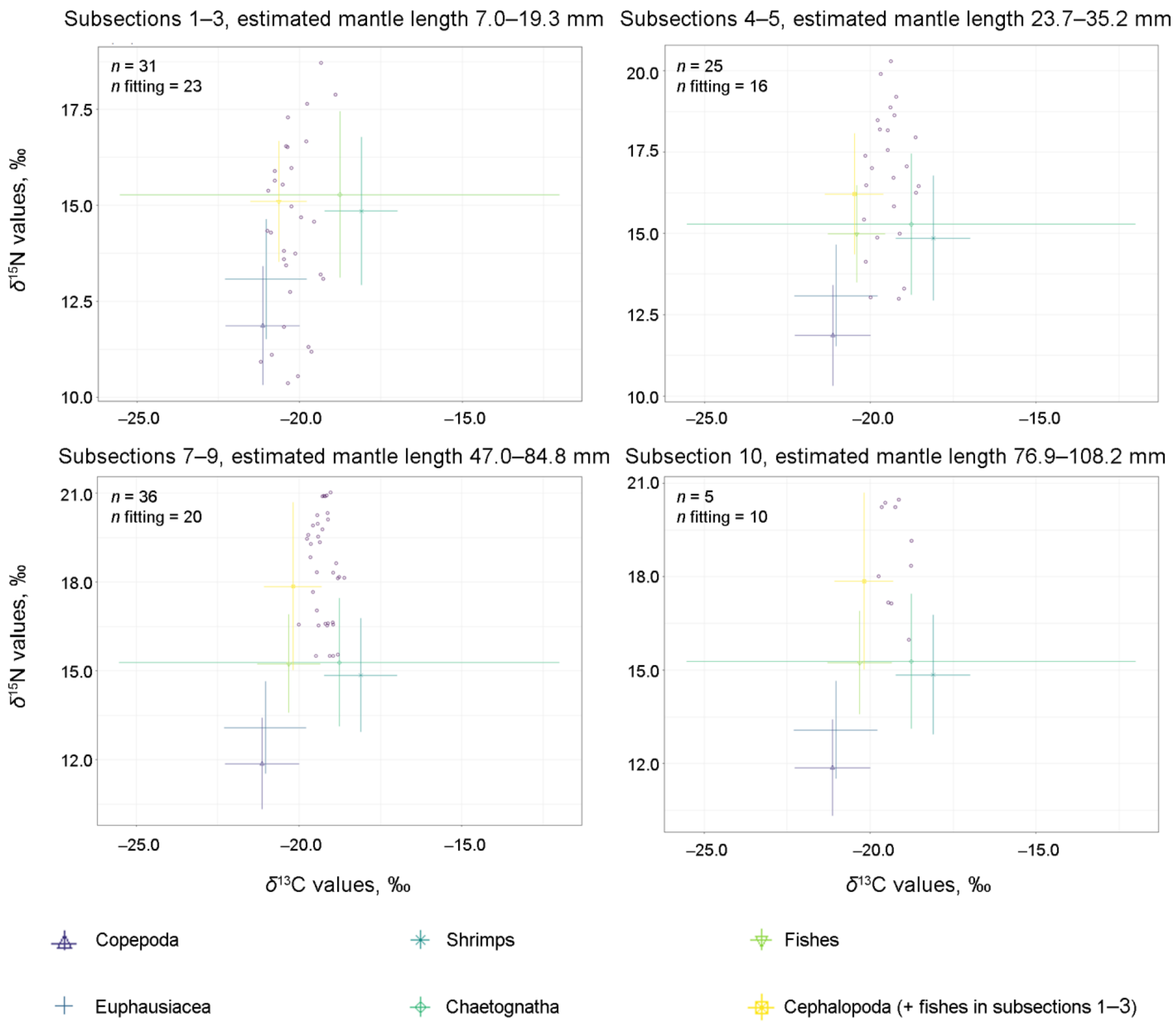

Figure S2. Isotopic niche overlap among the upper beak subsections of *Gonatus fabricii*, estimated in nicheROVER 1.1.0.

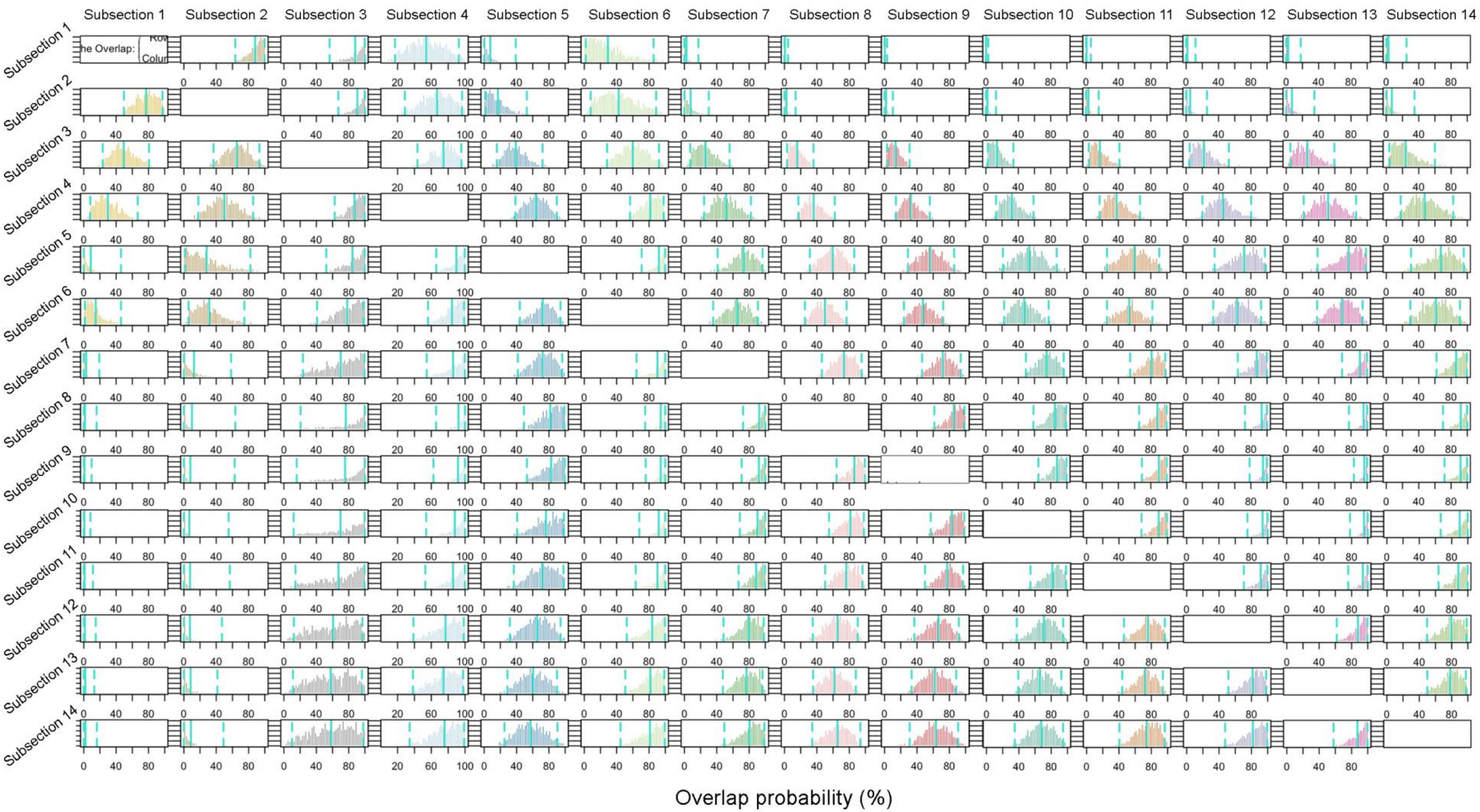

Supplement: Supplementary file 1 [file animals-12-03548-s001.zip › animals-1982426-supplementary.pdf]
